# Supplementary material for: AD-GCN: A novel graph convolutional network integrating multi-omics data for enhanced Alzheimer’s disease diagnosis
Source: PLoS One. 2025 Jun 2;20(6):e0325050. doi: 10.1371/journal.pone.0325050 (PMC12129192; doi:10.1371/journal.pone.0325050)
Supplement: S1 File — (DOCX) [file pone.0325050.s001.docx]

**S1 Fig. Heat map of LD values (at chromosome 19) and projection on 2D coordinates for genotype data at overlapping loci between the 1KGP European and ADNI White populations.**

**
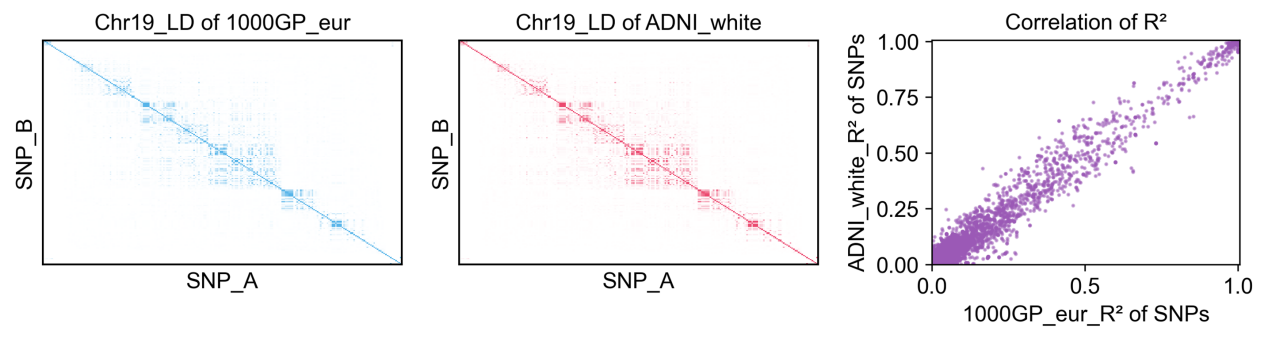
**

**S2 Fig. Linear fits based on LDpred PRS for 12 cognitive scores on CN vs. MCI.**

**
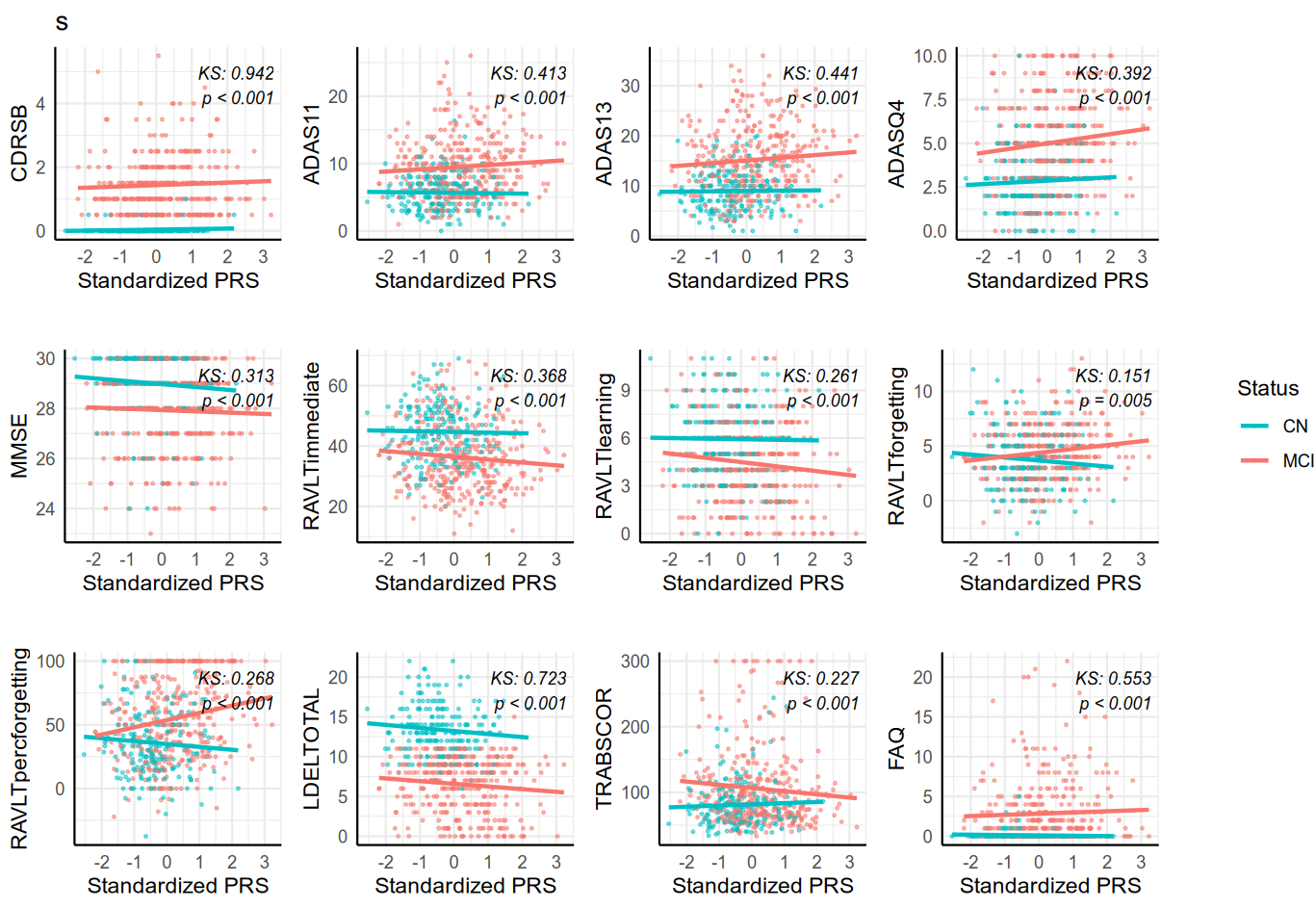
**

**S3 Fig. Linear fits based on LDpred PRS for 12 cognitive scores on MCI vs. AD.**

**
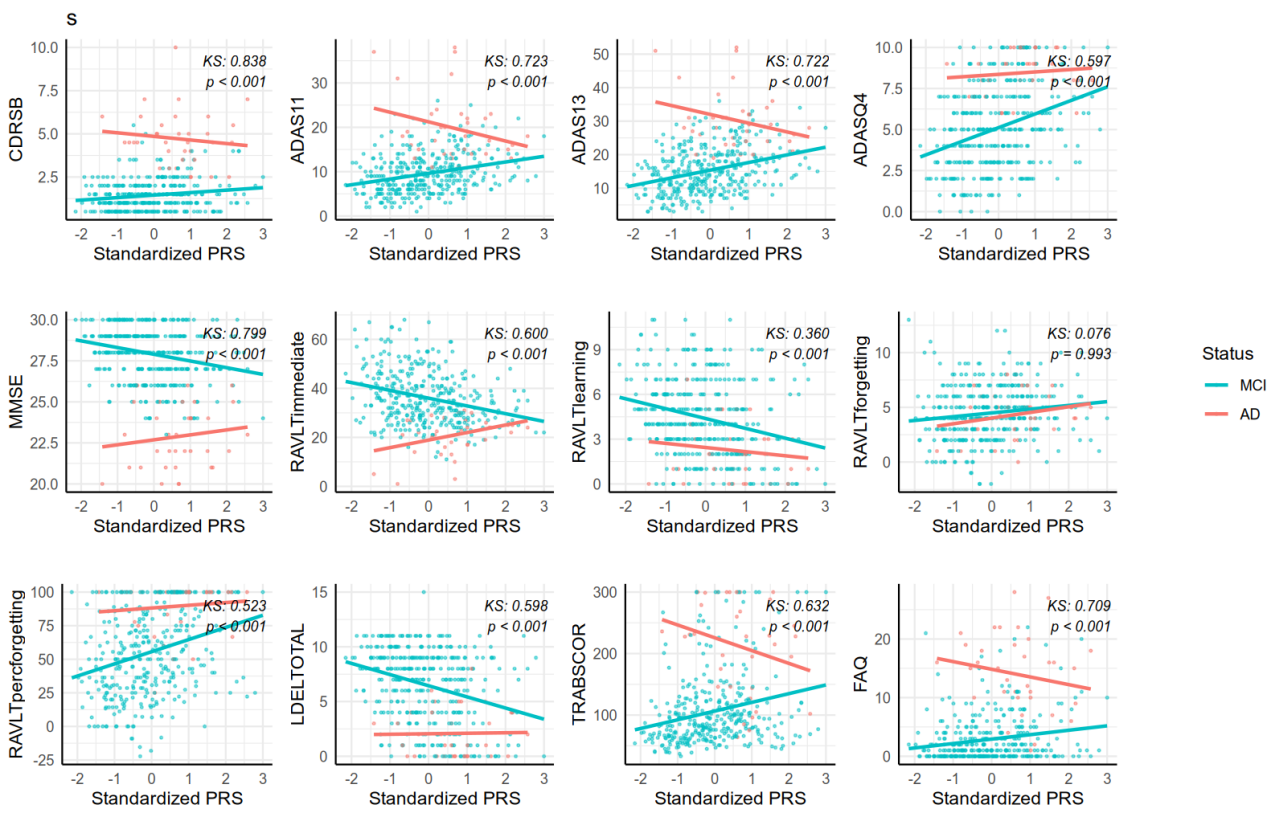
**

**S4 Fig. Linear fits based on LDpred PRS for 12 cognitive scores on CN vs. AD.**

**
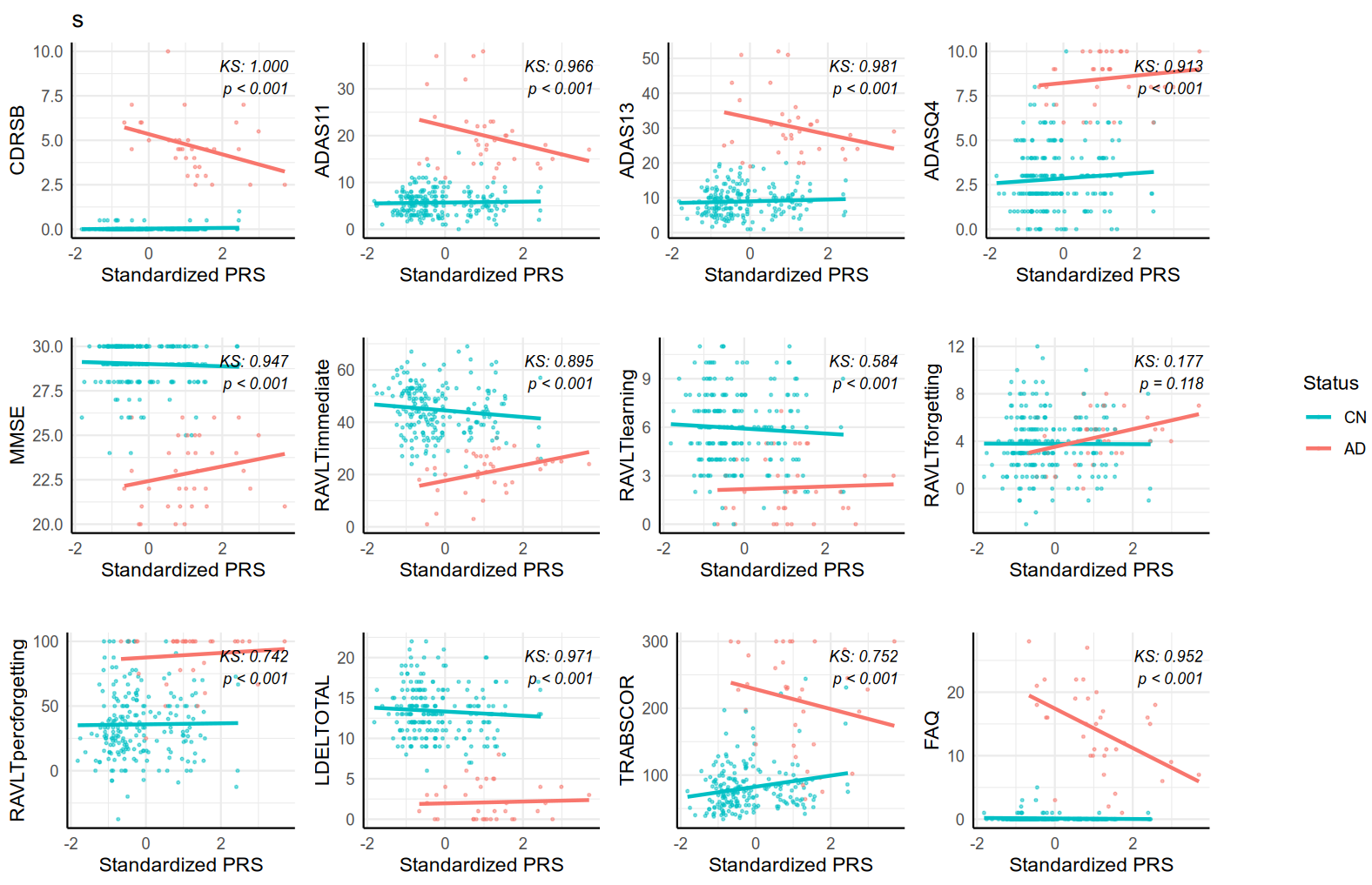
**

**S5 Fig. The distribution of Fisher scores in CN vs. MCI group.**


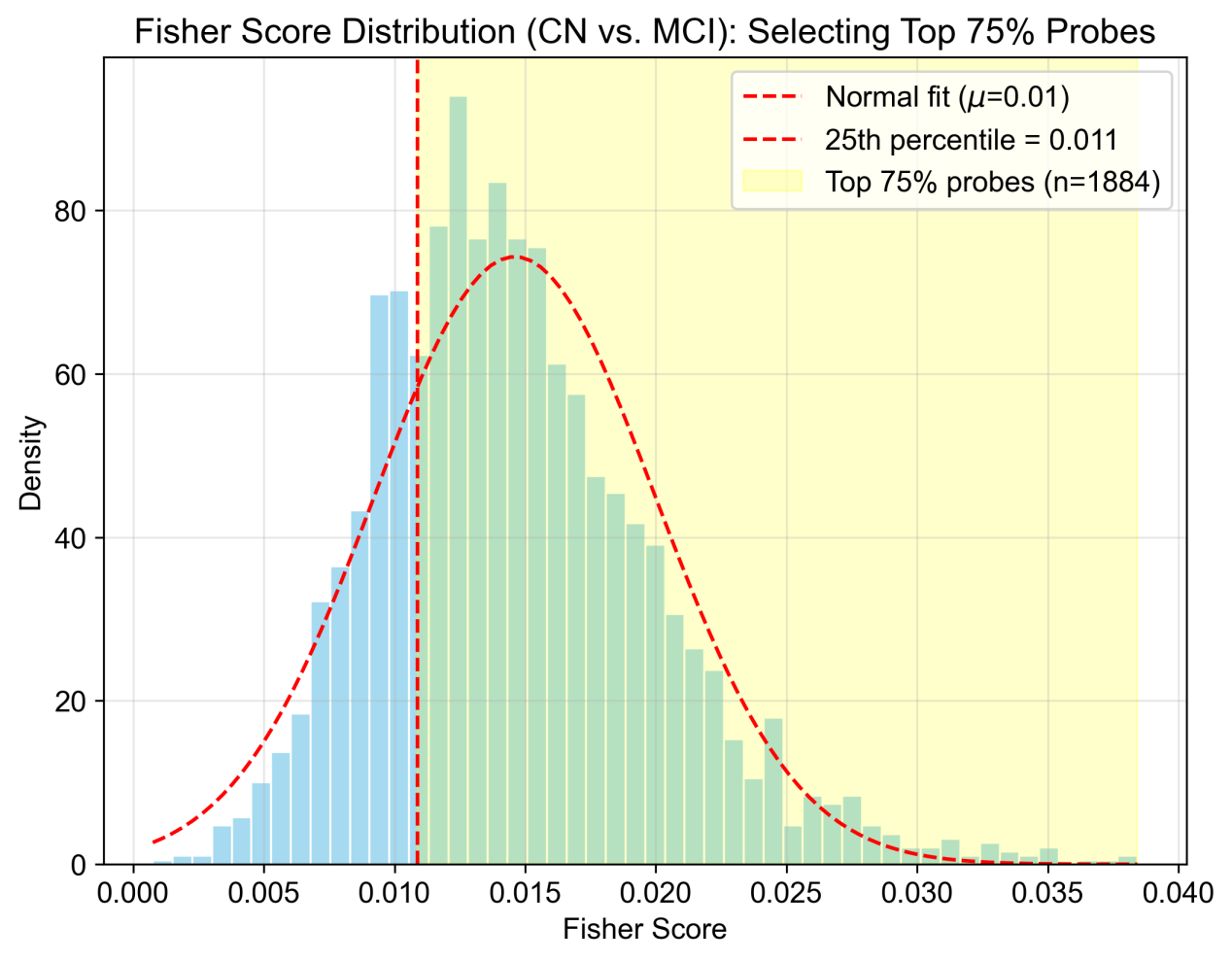


**S6 Fig. The distribution of Fisher scores in MCI vs. AD group.**


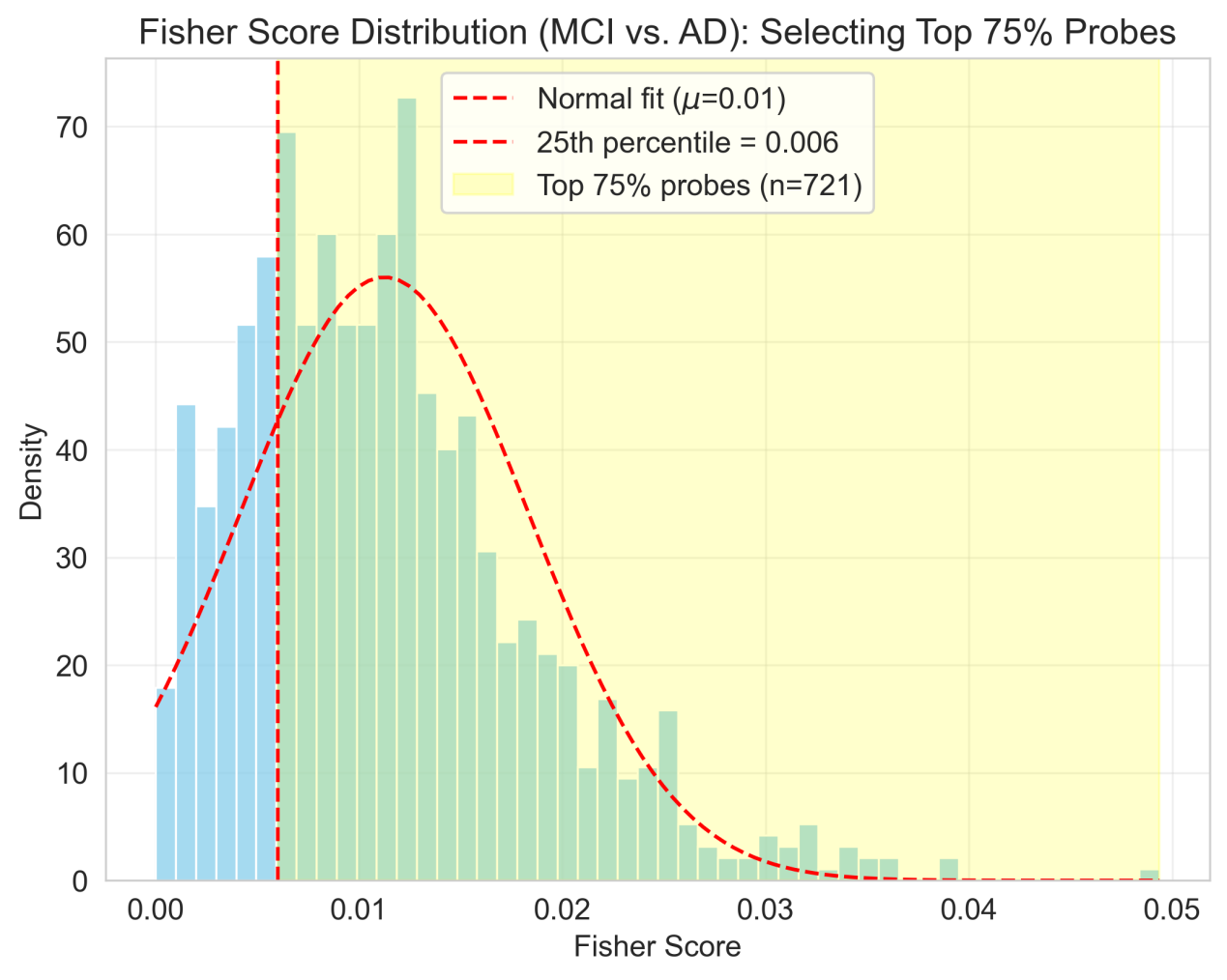


**S7 Fig. The distribution of Fisher scores in CN vs. AD group.**


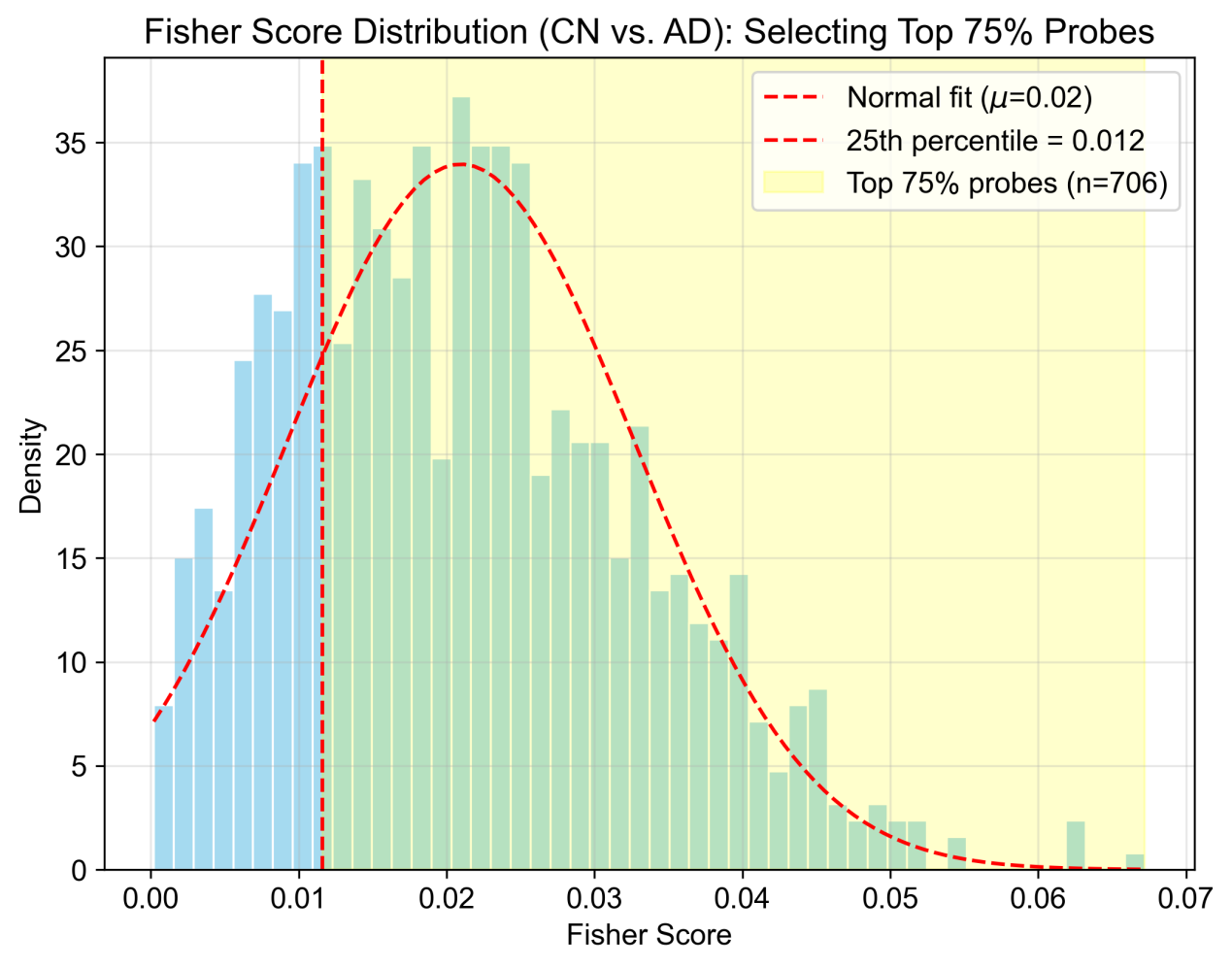


**S8 Fig. Comparison of AD-GCN ablation study results. Ablation experiments under three strategies all adopt a five-fold cross-validation approach.**


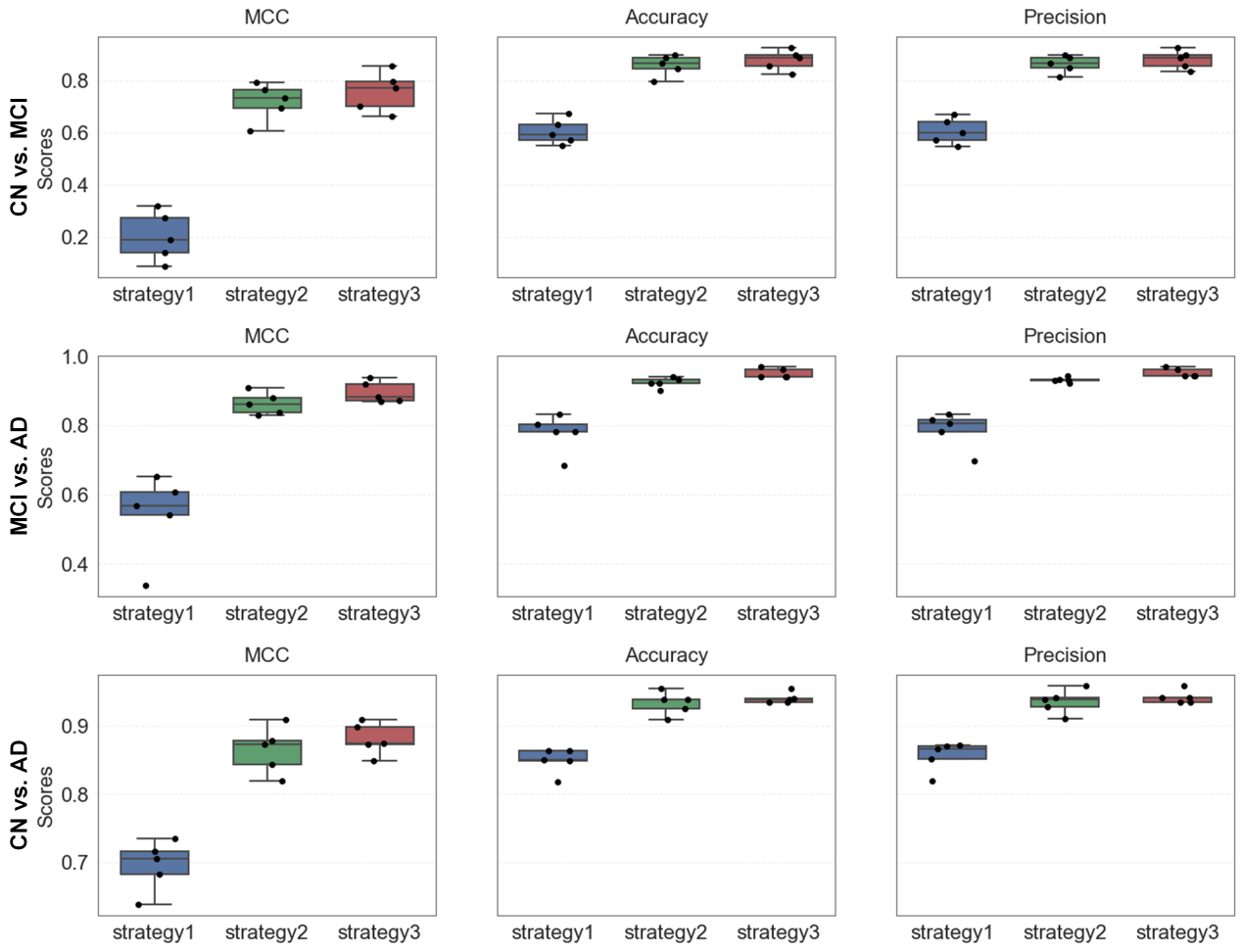


**S1 Table. Details of clinical cognitive assessment of ADNI data.**

| **Cognitive Assessment** | **Full Name** | **Description** |
| --- | --- | --- |
| **CDRSB** | Clinical Dementia Rating (Sum of Boxes) | The CDR-SB is a part of the Clinical Dementia Rating scale, used to evaluate the severity of dementia in six areas: memory, orientation, judgment and problem-solving, community affairs, home and hobbies, and personal care. Scores range from 0 to 18, with higher scores indicating more severe cognitive impairment. |
| **ADASQ4** | Alzheimer's Disease Assessment Scale (Question 4) | ADASQ4 assesses communication ability and speech content, specifically focusing on language impairment. It is part of the larger ADAS scale, which evaluates cognitive decline in Alzheimer's patients. |
| **ADAS11** | Alzheimer's Disease Assessment Scale (11-item Cognitive Subscale) | ADAS11 is used to measure cognitive function in Alzheimer's disease patients, assessing memory, language, and executive functioning. Higher scores indicate greater cognitive impairment. |
| **ADAS13** | Alzheimer's Disease Assessment Scale (13-item Cognitive Subscale) | ADAS13 is an extended version of ADAS11, adding two more items related to cognitive assessment. It provides a more detailed evaluation of cognitive impairment in Alzheimer's patients. |
| **MMSE** | Mini-Mental State Examination | The MMSE is a widely used tool for screening cognitive impairment, assessing areas such as memory, attention, language, and executive function. The total score is 30, with lower scores indicating more severe cognitive decline. |
| **RAVLTimmediate** | Rey Auditory Verbal Learning Test (Immediate Recall) | This test assesses the ability to recall a list of words immediately after hearing them, measuring short-term memory and immediate recall. |
| **RAVLTlearning** | Rey Auditory Verbal Learning Test (Learning) | This score evaluates the patient's ability to learn and retain words over multiple attempts, focusing on long-term memory and learning efficiency. |
| **RAVLTforgetting** | Rey Auditory Verbal Learning Test (Forgetting) | This score assesses how much information a patient forgets after learning, reflecting the retention and forgetting rate of long-term memory. |
| **RAVLTpercforgetting** | Rey Auditory Verbal Learning Test (Percent Forgetting) | This score calculates the percentage of words forgotten over time, indicating the severity of memory loss. |
| **LDELTOTAL** | Logical Memory Delayed Recall (Total Score) | This test measures a patient's ability to recall a specific story or information after a delay, assessing long-term memory retention. |
| **TRABSCOR** | Trail Making Test Part B (Total Score) | This cognitive test assesses executive functioning, attention, visual-motor speed, and cognitive flexibility. The score is based on the time it takes to complete the task, with higher times indicating worse performance. |
| **FAQ** | Functional Activities Questionnaire | The FAQ measures an individual's ability to perform daily activities independently, such as handling finances, shopping, cooking, and remembering appointments. Higher scores indicate more significant impairment in daily functioning. |

**S2 Table. LDpred PRS results in CN vs. MCI group.**

| **ID** | PRS | **ID** | PRS | **ID** | PRS | **ID** | PRS | **ID** | PRS |
| --- | --- | --- | --- | --- | --- | --- | --- | --- | --- |
| **0413** | 1.5246 | **2031** | 1.6908 | **1016** | 1.5045 | **4226** | 1.6304 | **2055** | 1.6899 |
| **0685** | 1.3498 | **4121** | 1.3939 | **1098** | 1.6819 | **4383** | 1.5564 | **2363** | 1.798 |
| **0729** | 1.738 | **4584** | 1.5053 | **1116** | 1.7418 | **4390** | 1.5171 | **4096** | 1.8348 |
| **1155** | 1.7783 | **0142** | 1.7146 | **4176** | 1.5878 | **4391** | 1.6739 | **4127** | 1.5848 |
| **1261** | 1.5573 | **2133** | 1.8046 | **4177** | 1.4078 | **4394** | 1.4512 | **0605** | 1.5531 |
| **1268** | 1.5226 | **2155** | 1.8243 | **4505** | 1.4014 | **4445** | 1.6452 | **0680** | 1.6564 |
| **2043** | 1.6323 | **2180** | 1.525 | **4508** | 1.4106 | **4462** | 1.5102 | **0709** | 1.7566 |
| **2073** | 1.4217 | **4313** | 1.6287 | **0156** | 1.6455 | **4465** | 1.8734 | **1187** | 1.8159 |
| **4171** | 1.7265 | **4349** | 1.4335 | **0555** | 1.7411 | **4522** | 1.6135 | **2407** | 1.5833 |
| **4213** | 1.5743 | **4399** | 1.5422 | **0997** | 1.388 | **4539** | 1.5536 | **4458** | 1.6197 |
| **4225** | 1.7104 | **4400** | 1.7989 | **4082** | 1.8076 | **4613** | 1.6219 | **4507** | 1.7073 |
| **4229** | 1.5816 | **4597** | 1.7265 | **4114** | 1.84 | **0089** | 1.7257 | **4514** | 1.7614 |
| **4237** | 1.3999 | **4285** | 1.6067 | **4414** | 1.7563 | **0746** | 1.5064 | **0112** | 1.6076 |
| **4262** | 1.5202 | **4293** | 1.6807 | **4464** | 1.7294 | **2153** | 1.5995 | **0259** | 1.5102 |
| **4270** | 1.5053 | **4367** | 1.6837 | **4582** | 1.726 | **2190** | 1.5189 | **0260** | 1.4679 |
| **4447** | 1.6506 | **1288** | 1.7122 | **0672** | 1.6306 | **2225** | 1.6477 | **0925** | 1.7169 |
| **4473** | 1.7327 | **0159** | 1.4308 | **0673** | 1.9175 | **2264** | 1.8202 | **1032** | 1.6153 |
| **4521** | 1.8389 | **0276** | 1.6415 | **0945** | 1.6565 | **4155** | 1.5273 | **1427** | 1.5839 |
| **4654** | 1.384 | **0337** | 1.7719 | **1023** | 1.6424 | **4216** | 1.7844 | **2213** | 1.4349 |
| **0907** | 1.2573 | **0626** | 1.4951 | **2378** | 1.8766 | **4259** | 1.7706 | **2234** | 1.7179 |
| **0908** | 1.6074 | **0984** | 1.7267 | **2380** | 1.9899 | **4300** | 1.4844 | **4148** | 1.6026 |
| **2374** | 1.4426 | **2100** | 1.4115 | **4430** | 1.6006 | **4311** | 1.4977 | **4197** | 1.6223 |
| **4081** | 1.6991 | **2124** | 1.3072 | **4491** | 1.4966 | **4312** | 1.8505 | **4198** | 1.6036 |
| **4119** | 1.4657 | **2125** | 1.4817 | **4562** | 2.0581 | **4360** | 1.5642 | **4210** | 1.843 |
| **4288** | 1.5976 | **2142** | 1.4403 | **0303** | 1.682 | **4382** | 1.5287 | **4301** | 1.6629 |
| **4350** | 1.6482 | **2150** | 1.7807 | **0454** | 1.5102 | **4393** | 1.5456 | **4604** | 1.8312 |
| **4354** | 1.5928 | **4254** | 1.5058 | **0467** | 1.3438 | **4443** | 1.7562 | **4645** | 1.5304 |
| **0448** | 1.5593 | **4276** | 1.6511 | **0501** | 1.6812 | **4552** | 1.8121 | **0135** | 1.4202 |
| **0546** | 1.6681 | **4335** | 1.6131 | **0566** | 1.6796 | **4559** | 1.5459 | **0138** | 1.3902 |
| **0553** | 1.4349 | **4402** | 1.6469 | **0588** | 1.8319 | **4614** | 1.5843 | **0205** | 1.5479 |
| **0572** | 1.7733 | **4421** | 1.5056 | **1078** | 1.9499 | **2099** | 1.4802 | **0225** | 1.4353 |
| **0602** | 1.6448 | **4659** | 1.7903 | **4015** | 1.5166 | **2121** | 1.5772 | **0229** | 1.5687 |
| **0610** | 1.533 | **0130** | 1.6653 | **4028** | 1.6758 | **2307** | 1.3936 | **0230** | 1.5109 |
| **2390** | 1.323 | **1097** | 1.5861 | **4030** | 1.7709 | **4090** | 1.5916 | **0272** | 1.4782 |
| **4168** | 1.6469 | **1351** | 1.9868 | **4071** | 1.4847 | **4208** | 1.514 | **0522** | 1.6073 |
| **4185** | 1.5052 | **2087** | 1.7664 | **4146** | 2.0184 | **4224** | 1.541 | **0545** | 1.5751 |
| **0498** | 1.7551 | **4173** | 1.5747 | **4214** | 1.569 | **4244** | 1.3521 | **0863** | 1.5751 |
| **0731** | 1.6083 | **4196** | 1.6489 | **4302** | 1.6488 | **4339** | 1.7428 | **1406** | 1.602 |
| **1130** | 1.6047 | **4320** | 1.5593 | **4308** | 1.5603 | **4428** | 1.5924 | **1408** | 1.6857 |
| **4150** | 1.587 | **4444** | 1.5361 | **4381** | 1.7897 | **2216** | 1.7796 | **2002** | 1.7804 |
| **4346** | 1.9019 | **0031** | 1.4809 | **4410** | 1.4741 | **2238** | 1.7061 | **2036** | 1.5932 |
| **4357** | 1.5212 | **0042** | 1.5779 | **4432** | 1.7377 | **2367** | 1.6986 | **2130** | 1.602 |

**Continue with S2 Table.**

| **4363** | 1.6469 | **0058** | 1.7238 | **0125** | 1.6106 | **4162** | 1.8346 | **2151** | 1.5721 |
| --- | --- | --- | --- | --- | --- | --- | --- | --- | --- |
| **4449** | 1.5687 | **0126** | 1.628 | **0679** | 1.5658 | **4234** | 1.7798 | **2220** | 1.6106 |
| **4515** | 1.7521 | **0217** | 1.3744 | **1010** | 1.624 | **4434** | 1.748 | **4553** | 1.5687 |
| **0101** | 1.8823 | **0331** | 1.9069 | **1425** | 1.4522 | **4503** | 1.4184 | **4571** | 1.8125 |
| **0128** | 1.8304 | **0625** | 1.6568 | **4004** | 1.5568 | **4560** | 1.5569 | **4653** | 1.4541 |
| **0698** | 1.7481 | **0887** | 1.6958 | **4014** | 1.4713 | **0160** | 1.7677 | **0778** | 1.7187 |
| **1206** | 1.5717 | **0926** | 1.3467 | **4037** | 1.4832 | **0171** | 1.4325 | **1246** | 1.7505 |
| **2106** | 1.5426 | **1046** | 1.8612 | **4041** | 1.3999 | **0172** | 1.5489 | **2332** | 1.5433 |
| **2394** | 2.0085 | **1190** | 1.4718 | **4051** | 1.5877 | **0269** | 1.7941 | **4220** | 1.5429 |
| **4272** | 1.5967 | **4020** | 1.6365 | **4060** | 1.4351 | **0667** | 1.7379 | **4369** | 1.5408 |
| **4387** | 1.6207 | **4035** | 1.4659 | **4138** | 1.4292 | **2047** | 1.7421 | **4371** | 1.7912 |
| **4467** | 1.9163 | **4115** | 1.7266 | **4143** | 1.6099 | **2052** | 1.597 | **4396** | 1.4703 |
| **4516** | 1.5293 | **4122** | 1.7352 | **4200** | 1.4539 | **4003** | 1.687 | **0285** | 1.8297 |
| **4611** | 1.7481 | **4164** | 1.7554 | **4271** | 1.8035 | **4018** | 1.6354 | **0289** | 1.6785 |
| **4620** | 1.5131 | **4243** | 1.5694 | **4510** | 1.8135 | **4050** | 1.5633 | **2391** | 1.6204 |
| **4637** | 1.5534 | **4448** | 1.5147 | **4513** | 1.8018 | **4275** | 1.585 | **2403** | 1.4257 |
| **0842** | 1.7118 | **4502** | 1.8445 | **1123** | 1.5891 | **4506** | 1.5595 | **4250** | 1.7459 |
| **1030** | 1.6581 | **0985** | 1.7422 | **1331** | 1.5894 | **0051** | 1.9605 | **4294** | 1.5388 |
| **2208** | 1.7874 | **2239** | 1.693 | **0671** | 1.8095 | **0352** | 1.4404 | **4352** | 1.4863 |
| **2381** | 1.858 | **4084** | 1.4414 | **0952** | 1.6316 | **2042** | 1.6394 | **4405** | 1.8589 |
| **4324** | 1.8191 | **4392** | 1.5837 | **0989** | 1.7628 | **2146** | 1.6327 | **4415** | 1.6925 |
| **4337** | 1.6884 | **0118** | 1.4798 | **1352** | 1.6121 | **2205** | 1.5584 | **4468** | 1.6567 |
| **4359** | 1.6612 | **0120** | 1.6109 | **2249** | 1.5473 | **4022** | 1.6465 | **4542** | 1.5597 |
| **4388** | 1.9368 | **0256** | 1.9143 | **4626** | 1.7921 | **4086** | 1.4273 | **4605** | 1.3899 |
| **4530** | 1.6312 | **0307** | 1.5023 | **0919** | 1.8034 | **4104** | 1.5405 | **0123** | 1.6179 |
| **4543** | 1.5725 | **0408** | 1.491 | **2357** | 1.6391 | **4157** | 1.4144 | **0384** | 1.5981 |
| **4612** | 1.6464 | **0835** | 1.7659 | **4557** | 1.4168 | **4202** | 1.5667 | **0441** | 1.4251 |
| **0002** | 1.5809 | **1045** | 1.4409 | **4578** | 1.5748 | **4205** | 1.3819 | **4281** | 1.4761 |
| **0008** | 1.3962 | **1387** | 1.6878 | **0934** | 1.5702 | **4463** | 1.9099 | **4309** | 1.8278 |
| **1080** | 1.4356 | **2219** | 1.6182 | **1269** | 1.5999 | **4480** | 1.8272 | **4356** | 1.5838 |
| **1282** | 1.379 | **2245** | 1.9822 | **2398** | 1.8865 | **4498** | 1.3084 | **4406** | 1.4206 |
| **2274** | 1.8912 | **2336** | 1.7119 | **0257** | 1.7416 | **4565** | 1.5781 | **4446** | 1.6227 |
| **4075** | 1.6791 | **0824** | 1.3638 | **2195** | 2.0862 | **0047** | 1.5089 | **4489** | 1.6107 |
| **4120** | 1.3339 | **0843** | 1.6592 | **2196** | 1.7774 | **0069** | 1.6446 | **4566** | 1.6131 |
| **4222** | 1.5767 | **0914** | 1.7596 | **2301** | 1.6479 | **1286** | 1.5017 | **4598** | 1.5956 |
| **4235** | 1.7538 | **1318** | 1.3782 | **2304** | 1.6801 | **4469** | 1.7473 | **0107** | 1.5311 |
| **4366** | 1.6924 | **2376** | 1.6409 | **4054** | 1.4637 | **4455** | 1.6986 | **0186** | 1.3499 |
| **4547** | 1.5288 | **2395** | 1.6688 | **4072** | 1.6387 | **4499** | 1.4991 | **4189** | 1.5659 |
| **0637** | 1.3451 | **4290** | 1.6683 | **4184** | 1.5098 | **0166** | 1.6253 | **4269** | 1.5623 |
| **4012** | 1.5609 | **4327** | 1.8375 | **4212** | 1.6733 | **0173** | 1.531 | **4433** | 1.671 |
| **4026** | 1.6159 | **4385** | 1.5431 | **4310** | 1.8828 | **0378** | 1.6632 | **0301** | 1.5136 |
| **4094** | 1.7275 | **0294** | 1.5509 | **0210** | 1.5595 | **0416** | 1.4331 | **0668** | 1.6696 |
| **4128** | 1.6767 | **0618** | 1.8168 | **2168** | 1.6211 | **1106** | 1.5349 | **0686** | 1.5754 |

**Continue with S2 Table.**

| **4188** | 1.7277 | **0867** | 1.6931 | **2184** | 1.5276 | **1118** | 1.6153 | **0722** | 1.6889 |
| --- | --- | --- | --- | --- | --- | --- | --- | --- | --- |
| **4545** | 1.4755 | **2018** | 1.4733 | **2187** | 1.7611 | **2392** | 1.6768 | **0800** | 1.904 |
| **4643** | 1.5389 | **2233** | 1.8504 | **2248** | 1.7757 | **0361** | 1.8594 | **0972** | 1.718 |
| **1186** | 1.582 | **4005** | 1.8658 | **2315** | 1.6433 | **0382** | 1.4184 | **1414** | 1.6252 |
| **4580** | 1.776 | **4029** | 1.9994 | **2316** | 1.6076 | **0649** | 1.4664 | **4299** | 1.5591 |
| **4595** | 1.5425 | **4032** | 1.5784 | **4061** | 1.6814 | **0834** | 1.8703 | **4331** | 1.6727 |
| **0169** | 1.7223 | **4042** | 1.8389 | **4067** | 1.7402 | **1232** | 1.4774 | **4351** | 1.6168 |
| **0519** | 1.5785 | **4203** | 1.6459 | **4174** | 1.5507 | **1243** | 1.7895 | **4466** | 1.5551 |
| **0520** | 1.7069 | **4218** | 1.6247 | **4217** | 1.5655 | **1249** | 1.6788 | **4482** | 1.4025 |
| **0548** | 1.7282 | **4474** | 1.4641 | **4332** | 1.7008 | **1271** | 1.7672 | **4520** | 1.6201 |
| **0557** | 1.7553 | **4590** | 1.6382 | **4340** | 1.4305 | **4010** | 1.5404 | **4596** | 1.4332 |
| **0558** | 1.4928 | **0214** | 1.9259 | **4424** | 1.7924 | **4043** | 1.4292 | **0697** | 1.63 |
| **0563** | 1.5646 | **0677** | 1.7209 | **4431** | 1.7038 | **4092** | 1.4482 | **1004** | 1.5788 |
| **0658** | 1.5722 | **0978** | 1.77 | **0315** | 1.6512 | **4167** | 1.598 | **1255** | 1.4914 |
| **2185** | 1.5371 | **1169** | 1.4482 | **2026** | 1.4056 | **4175** | 1.6676 | **4232** | 1.5912 |
| **2308** | 1.4932 | **2119** | 1.5551 | **2037** | 1.7746 | **4199** | 1.804 | **4426** | 1.632 |
| **4058** | 1.5659 | **2247** | 1.6405 | **2083** | 1.4095 | **4453** | 1.5591 | **2148** | 1.5742 |
| **4079** | 1.629 | **4386** | 1.3641 | **2093** | 1.8427 | **4483** | 1.4919 | **4077** | 1.5253 |
| **4080** | 1.4862 | **4429** | 1.5015 | **2116** | 1.7896 | **4635** | 1.7133 | **4372** | 1.4746 |
| **4093** | 1.5539 | **0514** | 1.5281 | **2164** | 1.4633 | **0072** | 1.7889 | **4621** | 1.644 |
| **4263** | 1.7516 | **0741** | 1.5659 | **4007** | 1.8039 | **0106** | 1.4702 | **2060** | 1.5468 |
| **4668** | 1.7802 | **0906** | 1.8892 | **4057** | 1.7184 | **0108** | 1.5687 | **4036** | 1.5141 |
| **0359** | 1.4382 | **0920** | 1.593 | **4102** | 1.5814 | **0113** | 1.5322 | **4100** | 1.4652 |
| **0702** | 1.6644 | **0922** | 1.9148 | **4131** | 1.5932 | **0298** | 1.4867 | **4187** | 1.7108 |
| **2007** | 1.3553 | **0923** | 1.4484 | **4206** | 1.6743 | **1300** | 1.4929 | **4255** | 1.6528 |
| **4292** | 1.5364 | **4365** | 1.6298 | **4376** | 1.4712 | **4420** | 1.5239 |  |  |

**S3 Table. LDpred PRS results in MCI vs. AD group.**

| **ID** | PRS | **ID** | PRS | **ID** | PRS | **ID** | PRS | **ID** | PRS |
| --- | --- | --- | --- | --- | --- | --- | --- | --- | --- |
| **0729** | 1.1112 | **4591** | 1.1168 | **4414** | 1.1007 | **4390** | 1.0552 | **4494** | 1.2254 |
| **1155** | 0.992 | **0142** | 1.1653 | **4582** | 1.1193 | **4394** | 1.0463 | **4507** | 1.1442 |
| **1268** | 1.1315 | **2133** | 1.1506 | **0673** | 1.2307 | **4445** | 1.0724 | **4514** | 1.1334 |
| **2043** | 1.1081 | **2155** | 1.1921 | **0945** | 1.0784 | **4462** | 1.043 | **4686** | 1.1403 |
| **2073** | 1.0107 | **2180** | 1.079 | **2378** | 1.0846 | **4465** | 1.1087 | **0112** | 1.0767 |
| **4171** | 1.0516 | **4597** | 1.0139 | **2380** | 1.1622 | **4522** | 1.0177 | **0925** | 1.0796 |
| **4229** | 1.0278 | **4696** | 1.1338 | **4430** | 1.0853 | **4539** | 1.0323 | **1032** | 1.1488 |
| **4237** | 1.0733 | **4252** | 1.1823 | **4562** | 1.1652 | **4613** | 0.995 | **1427** | 1.0658 |
| **4447** | 1.1145 | **4285** | 1.0478 | **0501** | 1.0729 | **0746** | 1.0392 | **2213** | 1.0852 |
| **4473** | 1.1214 | **4293** | 1.0527 | **0566** | 1.1357 | **2153** | 1.052 | **2234** | 0.9752 |
| **4521** | 1.1176 | **4477** | 1.1911 | **0588** | 1.1986 | **2190** | 1.0746 | **4197** | 1.1594 |
| **4654** | 1.0431 | **0276** | 0.9892 | **1078** | 1.219 | **2225** | 1.0145 | **4210** | 1.0872 |
| **0908** | 0.9875 | **0626** | 1.0552 | **4001** | 1.1052 | **2264** | 1.1116 | **4301** | 1.06 |
| **2374** | 1.0584 | **2100** | 1.1272 | **4015** | 1.1203 | **4216** | 0.9889 | **4500** | 1.1283 |
| **4136** | 1.0968 | **2124** | 1.0601 | **4030** | 1.1133 | **4259** | 1.0899 | **0135** | 1.064 |
| **4152** | 1.0053 | **2125** | 1.0783 | **4146** | 1.169 | **4300** | 1.0785 | **0138** | 1.0954 |
| **4354** | 1.0439 | **2142** | 1.0988 | **4214** | 1.0062 | **4311** | 1.0272 | **0205** | 1.0279 |
| **4373** | 1.1341 | **2150** | 1.1202 | **4302** | 1.0168 | **4312** | 1.0791 | **0225** | 1.0834 |
| **0448** | 1.0873 | **4402** | 1.0978 | **4381** | 1.0654 | **4360** | 0.9782 | **1406** | 0.9981 |
| **0546** | 1.1496 | **4659** | 1.2434 | **4432** | 1.0069 | **4443** | 1.0613 | **1408** | 1.0539 |
| **0572** | 1.2252 | **1097** | 1.0178 | **0679** | 1.0117 | **4614** | 1.0912 | **2002** | 1.0234 |
| **2390** | 1.0999 | **1351** | 1.2324 | **1010** | 1.1206 | **2099** | 1.0817 | **2036** | 1.0334 |
| **4168** | 1.1786 | **2087** | 1.1116 | **1425** | 1.06 | **2121** | 1.1129 | **2130** | 1.0447 |
| **4185** | 1.0792 | **4444** | 1.0662 | **4004** | 1.0552 | **2307** | 1.0504 | **2151** | 0.9935 |
| **4707** | 1.112 | **0042** | 1.03 | **4051** | 1.0275 | **4244** | 1.0847 | **2220** | 1.061 |
| **1130** | 1.143 | **0126** | 1.1644 | **4138** | 1.0761 | **2216** | 1.1308 | **4553** | 1.0343 |
| **4153** | 1.1181 | **0217** | 1.0835 | **4143** | 1.0158 | **2238** | 1.0187 | **4571** | 1.0849 |
| **4346** | 1.2021 | **0331** | 1.2033 | **4271** | 0.9605 | **2367** | 1.1399 | **4653** | 1.0603 |
| **4363** | 1.0503 | **0625** | 1.0854 | **4510** | 1.1415 | **4089** | 1.1542 | **1246** | 1.1409 |
| **4515** | 1.1248 | **0887** | 1.1602 | **4513** | 1.0927 | **4162** | 1.1866 | **2332** | 1.0116 |
| **4546** | 1.1021 | **1046** | 1.127 | **1331** | 1.0936 | **4434** | 1.0349 | **4220** | 1.0471 |
| **0101** | 1.2047 | **4035** | 1.1301 | **0671** | 1.1146 | **0160** | 1.1485 | **0285** | 1.1083 |
| **0128** | 1.1699 | **4115** | 1.036 | **0952** | 1.1358 | **0269** | 1.1058 | **0289** | 1.0169 |
| **0698** | 1.1364 | **4122** | 1.0177 | **0989** | 1.0999 | **0667** | 1.0983 | **2391** | 1.128 |
| **2106** | 1.0841 | **4243** | 1.068 | **1352** | 1.0911 | **2047** | 1.1494 | **2403** | 1.0516 |
| **2394** | 1.191 | **4501** | 1.1537 | **2249** | 1.0401 | **2052** | 1.059 | **4250** | 1.1254 |
| **4272** | 1.0331 | **4502** | 1.1549 | **4626** | 1.0208 | **4201** | 1.1834 | **4294** | 1.1311 |
| **4467** | 1.193 | **2239** | 1.1151 | **0919** | 1.0718 | **4215** | 1.1491 | **4405** | 1.1215 |
| **4568** | 1.1298 | **4223** | 1.043 | **2357** | 1.0187 | **0051** | 1.1822 | **4415** | 1.235 |
| **4611** | 1.0789 | **4280** | 1.1507 | **4557** | 1.1061 | **2042** | 1.0314 | **4468** | 1.0091 |
| **1030** | 1.0111 | **4392** | 1.0833 | **1269** | 1.0084 | **2146** | 1.0006 | **4542** | 1.2348 |
| **2208** | 1.0999 | **0256** | 1.2024 | **2398** | 1.2287 | **2205** | 1.1085 | **4589** | 1.2233 |

**Continue with S3 Table.**

| **2381** | 1.1959 | **0307** | 1.0478 | **2195** | 1.1924 | **4022** | 1.1655 | **4605** | 1.0733 |
| --- | --- | --- | --- | --- | --- | --- | --- | --- | --- |
| **4324** | 1.0724 | **0408** | 1.0835 | **2196** | 1.1242 | **4157** | 1.0788 | **0384** | 1.0805 |
| **4359** | 1.1075 | **0835** | 1.1258 | **2301** | 1.026 | **4202** | 1.0194 | **4281** | 1.0669 |
| **4530** | 1.1112 | **1045** | 1.0732 | **2304** | 0.9714 | **4205** | 1.0713 | **4309** | 1.1005 |
| **4543** | 1.0476 | **1387** | 1.1784 | **4054** | 1.0037 | **4463** | 1.1187 | **4356** | 1.0162 |
| **1080** | 1.0667 | **2219** | 1.0412 | **4072** | 1.0175 | **4480** | 1.1116 | **4406** | 1.058 |
| **1282** | 1.0622 | **2245** | 1.1505 | **4184** | 1.017 | **4498** | 1.0613 | **4489** | 1.0343 |
| **2274** | 1.1072 | **2336** | 1.1458 | **4212** | 0.9897 | **4565** | 1.0863 | **4657** | 1.1286 |
| **4235** | 1.0101 | **0914** | 1.14 | **4310** | 1.0936 | **4455** | 1.001 | **4676** | 1.1604 |
| **4366** | 1.1067 | **1318** | 1.0741 | **2168** | 1.0305 | **0378** | 1.1246 | **0107** | 0.9932 |
| **4547** | 1.0682 | **2376** | 1.1735 | **2184** | 1.1268 | **1106** | 1.0429 | **4189** | 1.1326 |
| **4012** | 1.0507 | **2395** | 1.1255 | **2187** | 1.0942 | **1118** | 1.086 | **0668** | 1.0717 |
| **4094** | 1.0337 | **4307** | 1.1441 | **2248** | 1.1548 | **2392** | 1.0012 | **0722** | 1.0586 |
| **4128** | 1.1437 | **4327** | 1.2627 | **2315** | 1.0474 | **0361** | 1.1341 | **0800** | 1.2218 |
| **4188** | 1.1541 | **0294** | 1.0774 | **2316** | 1.1387 | **0649** | 1.1408 | **1414** | 1.1395 |
| **1186** | 1.102 | **0867** | 1.0412 | **4061** | 1.0989 | **0834** | 1.1038 | **4211** | 1.0976 |
| **4595** | 1.0621 | **2018** | 1.0654 | **4067** | 1.0283 | **1243** | 1.1474 | **4258** | 1.1539 |
| **0169** | 1.1196 | **2233** | 1.0798 | **4217** | 1.0656 | **1271** | 1.1031 | **4299** | 1.07 |
| **0557** | 1.0926 | **4005** | 1.1416 | **4332** | 1.0325 | **4167** | 1.1327 | **4331** | 1.1299 |
| **0563** | 1.0745 | **4024** | 1.0585 | **4431** | 1.0381 | **4175** | 1.1282 | **4351** | 1.0969 |
| **0658** | 1.0251 | **4029** | 1.1635 | **4692** | 1.2495 | **4199** | 1.0049 | **4596** | 1.0329 |
| **2185** | 1.0614 | **4042** | 0.9837 | **2026** | 1.0819 | **4338** | 1.07 | **4672** | 1.1885 |
| **2308** | 1.0633 | **4203** | 1.1325 | **2037** | 1.109 | **4635** | 1.1473 | **0697** | 1.0625 |
| **4039** | 1.0508 | **4590** | 1.0159 | **2083** | 1.1162 | **0108** | 1.1354 | **1004** | 1.1285 |
| **4058** | 1.1728 | **0214** | 1.163 | **2093** | 1.0729 | **1300** | 1.0758 | **1255** | 1.0499 |
| **4079** | 1.1072 | **0978** | 1.1364 | **2116** | 0.9856 | **2055** | 1.1373 | **4232** | 1.0399 |
| **4263** | 1.106 | **2119** | 1.0361 | **2164** | 1.0421 | **2363** | 1.014 | **4426** | 1.058 |
| **4615** | 1.0764 | **2247** | 1.0005 | **4007** | 1.1281 | **4096** | 1.2755 | **2148** | 1.0172 |
| **4668** | 1.099 | **0514** | 1.0425 | **4057** | 1.156 | **4127** | 1.0217 | **4077** | 1.0454 |
| **0702** | 1.1797 | **0906** | 1.1131 | **4102** | 1.0387 | **4526** | 1.2184 | **4621** | 1.0363 |
| **2007** | 1.0808 | **0922** | 1.144 | **4131** | 1.1592 | **0709** | 1.1982 | **2060** | 1.0455 |
| **2031** | 1.0412 | **1116** | 1.2495 | **4206** | 1.033 | **1187** | 1.1304 | **4036** | 1.0352 |
| **4353** | 1.0643 | **0997** | 1.0782 | **4226** | 1.0295 | **2407** | 1.0223 | **4187** | 0.9772 |
| **4584** | 1.1435 | **4114** | 1.0727 | **4383** | 1.013 | **4458** | 1.1393 | **4420** | 1.0899 |
| **0729** | 1.1112 | **4591** | 1.1168 | **4414** | 1.1007 | **4390** | 1.0552 |  |  |

**S4 Table. LDpred PRS results in CN vs. AD group.**

| **ID** | PRS | **ID** | PRS | **ID** | PRS | **ID** | PRS | **ID** | PRS |
| --- | --- | --- | --- | --- | --- | --- | --- | --- | --- |
| **0413** | 1.0443 | **4039** | 1.2798 | **4218** | 1.1241 | **4393** | 1.0352 | **0259** | 1.0487 |
| **0685** | 1.1313 | **4080** | 0.9645 | **4474** | 1.098 | **4552** | 1.3019 | **0260** | 0.9973 |
| **1261** | 1.0261 | **4093** | 1.0013 | **0677** | 1.0607 | **4559** | 0.8524 | **4148** | 0.9971 |
| **4213** | 1.148 | **4615** | 1.0695 | **1169** | 1.005 | **4090** | 0.9812 | **4198** | 1.1901 |
| **4225** | 1.3193 | **0359** | 0.9643 | **4386** | 1.0157 | **4208** | 1.0571 | **4500** | 1.0372 |
| **4262** | 0.9951 | **4121** | 1.1039 | **4429** | 1.1042 | **4224** | 0.9469 | **4604** | 1.3535 |
| **4270** | 1.3228 | **4353** | 1.2901 | **0741** | 1.244 | **4339** | 1.5371 | **4645** | 1.0253 |
| **0907** | 0.9672 | **4591** | 1.2606 | **0920** | 1.3009 | **4428** | 1.1368 | **0229** | 1.1995 |
| **4081** | 1.2511 | **4313** | 1.3303 | **0923** | 1.0937 | **4089** | 1.4195 | **0230** | 1.1016 |
| **4119** | 1.0753 | **4349** | 1.0858 | **1016** | 1.0786 | **4234** | 1.3851 | **0272** | 0.9889 |
| **4136** | 1.2311 | **4399** | 1.2408 | **1098** | 1.3107 | **4503** | 0.9299 | **0522** | 1.1338 |
| **4152** | 1.1077 | **4400** | 1.3953 | **4176** | 1.2496 | **4560** | 0.9347 | **0545** | 1.0619 |
| **4288** | 1.0169 | **4696** | 1.2997 | **4177** | 1.0428 | **0171** | 0.9684 | **0863** | 1.1216 |
| **4350** | 1.1335 | **4252** | 1.2765 | **4505** | 0.9887 | **0172** | 0.9882 | **0778** | 1.3565 |
| **4373** | 1.2604 | **4367** | 1.2491 | **4508** | 1.1078 | **4003** | 1.2508 | **4369** | 0.9691 |
| **0553** | 1.0059 | **4477** | 1.3311 | **0156** | 1.0104 | **4018** | 1.1007 | **4371** | 1.3907 |
| **0602** | 0.9448 | **1288** | 0.9282 | **0555** | 1.3415 | **4050** | 0.9732 | **4396** | 1.0139 |
| **0610** | 1.0469 | **0159** | 1.003 | **4082** | 1.3458 | **4201** | 1.5245 | **4352** | 1.0892 |
| **4707** | 1.3957 | **0337** | 1.2723 | **4464** | 1.3516 | **4215** | 1.4282 | **4589** | 1.5353 |
| **0498** | 1.3051 | **0984** | 1.3375 | **0672** | 0.9493 | **4275** | 1.0487 | **0123** | 1.0654 |
| **0731** | 1.1323 | **4254** | 1.0553 | **1023** | 1.2347 | **4506** | 1.0091 | **0441** | 1.1158 |
| **4150** | 1.1019 | **4276** | 1.3139 | **4491** | 1.1382 | **0352** | 0.9994 | **4446** | 1.1061 |
| **4153** | 1.3916 | **4335** | 1.0233 | **0303** | 1.3257 | **4086** | 1.0283 | **4566** | 1.3898 |
| **4357** | 1.0138 | **4421** | 1.0251 | **0454** | 1.0041 | **4104** | 1.0706 | **4598** | 0.8852 |
| **4449** | 1.0524 | **0130** | 1.1043 | **0467** | 1.0669 | **0047** | 1.0925 | **4657** | 1.2273 |
| **4546** | 1.3128 | **4173** | 1.0608 | **4001** | 1.1012 | **0069** | 1.0567 | **4676** | 1.3642 |
| **1206** | 1.0662 | **4196** | 1.1462 | **4028** | 0.9842 | **1286** | 0.9977 | **0186** | 0.9996 |
| **4387** | 1.2213 | **4320** | 1.0286 | **4071** | 1.0656 | **4469** | 1.2719 | **4269** | 0.9745 |
| **4516** | 1.0938 | **0031** | 0.9884 | **4308** | 0.9671 | **4499** | 1.1555 | **4433** | 1.238 |
| **4568** | 1.355 | **0058** | 1.2979 | **4410** | 1.1149 | **0166** | 1.1997 | **0301** | 1.0457 |
| **4620** | 1.0203 | **0926** | 1.0716 | **0125** | 1.0635 | **0173** | 1.0743 | **0686** | 1.0241 |
| **4637** | 1.0394 | **1190** | 1.0546 | **4014** | 1.1323 | **0416** | 1.0049 | **0972** | 1.305 |
| **0842** | 1.3593 | **4020** | 1.0058 | **4037** | 1.0071 | **0382** | 0.9576 | **4211** | 1.1443 |
| **4337** | 1.1108 | **4164** | 1.135 | **4041** | 0.9588 | **1232** | 1.0536 | **4258** | 1.3472 |
| **4388** | 1.5351 | **4448** | 1.1375 | **4060** | 0.9749 | **1249** | 1.2634 | **4466** | 1.1329 |
| **4612** | 1.2816 | **4501** | 1.3059 | **4200** | 0.9455 | **4010** | 1.0047 | **4482** | 1.0096 |
| **0002** | 1.1037 | **0985** | 1.2293 | **1123** | 1.3177 | **4043** | 0.9628 | **4520** | 1.0769 |
| **0008** | 0.863 | **4084** | 1.0517 | **4578** | 1.0584 | **4092** | 1.1013 | **4672** | 1.7356 |
| **4075** | 1.2774 | **4223** | 1.0692 | **0934** | 1.1542 | **4338** | 1.1127 | **4372** | 1.036 |
| **4120** | 1.0171 | **4280** | 1.3146 | **0257** | 1.3765 | **4453** | 1.0439 | **4100** | 1.1022 |
| **4222** | 1.2223 | **0118** | 1.0087 | **0210** | 0.9835 | **4483** | 1.0933 | **4255** | 1.3176 |
| **0637** | 0.9118 | **0120** | 1.2614 | **4174** | 1.2386 | **0072** | 1.1832 | **4292** | 1.163 |

**Continue with S4 Table.**

| **4026** | 1.0258 | **0824** | 1.0431 | **4340** | 1.0662 | **0106** | 1.0037 | **4365** | 1.1332 |
| --- | --- | --- | --- | --- | --- | --- | --- | --- | --- |
| **4545** | 0.9649 | **0843** | 1.0186 | **4424** | 1.3494 | **0113** | 0.9724 | **4376** | 1.0097 |
| **4643** | 1.0049 | **4290** | 1.2923 | **4692** | 1.5562 | **0298** | 1.116 |  |  |
| **4580** | 1.5295 | **4307** | 1.3416 | **0315** | 1.0525 | **4526** | 1.5854 |  |  |
| **0519** | 1.034 | **4385** | 1.2428 | **4391** | 1.0626 | **0605** | 1.2487 |  |  |
| **0520** | 1.527 | **0618** | 1.335 | **0089** | 1.0383 | **0680** | 1.0688 |  |  |
| **0548** | 1.323 | **4024** | 1.2718 | **4155** | 1.2454 | **4494** | 1.6216 |  |  |

**S5 Table. Number of probes in information entropy screening process.**

| **Group** | **information Gain > 0** | **Quartile (25%~100%)** |
| --- | --- | --- |
| CN vs. MCI | 1,165 | 1,161 |
| MCI vs. AD | 426 | 422 |
| CN vs.AD | 478 | 472 |

**S6 Table.** **Numbers of methylation probes selected after quality control.**

| **Condition** | **CN vs. MCI** | **MCI vs. AD** | **CN vs. AD** |
| --- | --- | --- | --- |
| Probes processed by ChAMP | 6,145 | 7,442 | 6,085 |
| Probes on CpG islands | 2,512 | 961 | 942 |
| Probes filtered by Fisher Score | 1,884 | 720 | 706 |
| Probes filtered by entropy | 1,161 | 422 | 472 |

**S7 Table. Top 10 methylation features selected by filtering and embedding methods.**

| **Group** | **Top10 probes** |
| --- | --- |
| CN vs. MCI | cg17750572, cg01452847, cg13348062, cg18627235, cg15452204, cg01821149, cg00884606, cg03354992, cg09173768, cg14515364 |
| MCI vs. AD | cg14718065, cg09440270, cg06980531, cg04143909, cg08026735, cg16664778, cg05313129, cg02168442, cg22832802, cg15802263 |
| CN vs. AD | cg26896946, cg06536614, cg07972135, cg27657429, cg18678645, cg24199400, cg04481923, cg11921736, cg05652809, cg01755562 |

**S8 Table. Evaluation results of different ML integrative methods.**

| **Score** | | **Stategy** | **CN vs. MCI** | | | **MCI vs. AD** | | | **CN vs. AD** | | |
| --- | --- | --- | --- | --- | --- | --- | --- | --- | --- | --- | --- |
|  |  |  | **CI** | **TI** | **MI** | **CI** | **TI** | **MI** | **CI** | **TI** | **MI** |
| MCC | C | | 0.23 | 0.13 | 0.19 | 0.43 | 0.42 | 0.49 | 0.49 | 0.43 | 0.52 |
|  | C+P | | 0.37 | 0.28 | 0.41 | 0.50 | 0.45 | 0.50 | 0.62 | 0.57 | 0.70 |
|  | C+P+M | | 0.53 | 0.48 | 0.58 | 0.56 | 0.51 | 0.69 | 0.76 | 0.75 | 0.78 |
| ACC | C | | 0.62 | 0.57 | 0.61 | 0.74 | 0.74 | 0.76 | 0.78 | 0.75 | 0.79 |
|  | C+P | | 0.68 | 0.64 | 0.70 | 0.79 | 0.77 | 0.80 | 0.83 | 0.81 | 0.87 |
|  | C+P+M | | 0.77 | 0.74 | 0.79 | 0.83 | 0.82 | 0.88 | 0.89 | 0.89 | 0.90 |
| PRE | C | | 0.65 | 0.60 | 0.62 | 0.65 | 0.66 | 0.67 | 0.72 | 0.65 | 0.75 |
|  | C+P | | 0.73 | 0.68 | 0.74 | 0.69 | 0.66 | 0.72 | 0.76 | 0.73 | 0.80 |
|  | C+P+M | | 0.80 | 0.77 | 0.79 | 0.70 | 0.70 | 0.88 | 0.82 | 0.82 | 0.90 |

*Abbreviations:* *CI* Concatenation-based integration, TI Transformation-based integration, *MI* Model-based integration, *MCC* Matthews correlation coefficient, *ACC* Accuracy, *PRE* Precision. The CI and TI methods utilize the average scores from multiple classifiers as evaluation metrics. MI approaches employ the mean of five-fold cross-validation results as the assessment criterion.

**S9 Table. Comparison of computing requirements of ML integrative methods and AD-GCN model diagnostics.**

| **Group** | **Model** | **Runtime** | **Peak memory** |
| --- | --- | --- | --- |
| CN vs. MCI | CI  TI  MI  AD-GCN | 3.28 s | 168.64 MiB |
|  |  | 2.75 s  5.49 s | 170.17 MiB  175.95 MiB |
|  |  | 13.2 s | 302.14 MiB |
| MCI vs. AD | CI  TI  MI  AD-GCN | 2.48 s | 165.83 MiB |
|  |  | 2.35 s  3.99 s | 168.89 MiB  179.60 MiB |
|  |  | 11.2 s | 303.00 MiB |
| CN vs. AD | CI  TI  MI  AD-GCN | 2.2 s | 166.07 MiB |
|  |  | 2.05 s  3.9 s | 166.65 MiB  168.36 MiB |
|  |  | 13.5 s | 290.98 MiB |

*Abbreviations:* *CI* Concatenation-based integration, TI Transformation-based integration, *MI* Model-based integration.
