# Supplementary material for: AD-GCN: A novel graph convolutional network integrating multi-omics data for enhanced Alzheimer’s disease diagnosis
Source: PLoS One. 2025 Jun 2;20(6):e0325050. doi: 10.1371/journal.pone.0325050 (PMC12129192; doi:10.1371/journal.pone.0325050)
Supplement: Attachment — Submitted filename: Response to Reviewers.docx [file pone.0325050.s003.docx]

Ref: Manuscript Number: PONE-D-25-05441

Title: **AD-GCN: A novel graph convolutional network integrating multi-omics data for enhanced Alzheimer’s disease diagnosis**

Dear Editors and Reviewers:

We appreciate you and the reviewers for your precious time in reviewing our paper and providing valuable comments. It was your valuable and insightful comments that led to possible improvements in the current version. I would like to express my sincere gratitude for your time and effort in handling my manuscript, “AD-GCN: A novel graph convolutional network integrating multi-omics data for enhanced Alzheimer’s disease diagnosis”, and for providing me with the opportunity to revise it. I have completed the major revision of the manuscript in strict accordance with the reviewers’ requirements.

For each reviewer’s comments, I have provided detailed and respectful responses. I have carefully addressed each and every comment in a point- by-point manner and made all the necessary adjustments to the manuscript.

Moreover, I stated that the funders had no role: The funders had no role in study design, data collection and analysis, decision to publish, or preparation of the manuscript. I have added a figure label and title for Figures 1 to 11 in my main manuscript. And I have added Supporting Information captions at the end of my manuscript, and updated all in-text citations to match accordingly.

**Reviewer #1**

**Comment 1:** *The english text sounds well in all paper sections, the topic and idea of the study original and absolutely current. Methodology applied is correct as well as statistic analysis. I only suggest to better summarize and focus the introduction around the study question.*

**Author Response 1:** We sincerely appreciate your positive assessment of our manuscript’s originality, methodology, and statistical rigor. In response to your valuable suggestion to better summarize the Introduction Section, we have implemented the following targeted revisions: (1) included a small introduction about what AD is and an epidemiology of AD in China (**Lines 58-62 in Manuscript**); (2) deleted sentences with little relevance (“*ABCA7* and *CLU*. The *APOE ɛ4* allele is...”); (3) the methodological background has been revised to clarify the positioning of current work within existing machine learning ensemble and GCN-based multi-omics integration approaches (**Lines 83-104 in Manuscript**).

**Reviewer #2**

**Comment 1** [Abstract-1]**:** *Line 37-38: multi-omics risk factors, such as age, sex, education level, and genomic and epigenomic influences. This should be changed, as when I think of multi-omics I think of genomics, transcriptomics etc, and not of age, sex, etc.*

**Author Response 1:** We agree that “multi-omics” should refer specifically to biological data types such as genomics, transcriptomics, and epigenomics, while demographic factors like age, sex, and education level should be categorized separately. We have revised the sentence accordingly to clarify this distinction (**Lines 36-38 in Manuscript**):

“Alzheimer’s disease (AD) etiology is complex, influenced by demographic risk factors such as age, sex, and educational level, alongside multi-omics factors derived from genomics, transcriptomics, and epigenomics.”

**Comment 2** [Introduction-1]**:** *The authors should include a small introduction about what AD is. An epidemiology of AD in China if this information is possible.*

**Author Response 2:** We have added a concise introduction to Alzheimer’s disease, including its definition and epidemiology in China, to provide context for the study [1]. This addition highlights the importance of our research in addressing this public health challenge (**Lines 58-62 in Manuscript**).

Reference:

[1] Longfei J, Yifeng D, Lan C, et al. Prevalence, risk factors, and management of

dementia and mild cognitive impairment in adults aged 60 years or older in China: a crosssectional study[J]. The Lancet Public Health, 2020, 5(12):661-671.

**Comment 3** [Introduction-2]**:** *Line 96: Contributions should be changed to aims.*

**Author Response 3:** We have revised the “contributions” section to “aims” to better align with the following content.

**Comment 4** [Methods-1]**:** *Line 108. Remove from the ADNI.*

**Author Response 4:** We have removed the phrase “from the ADNI” from Line 108 to improve clarity and conciseness.

**Comment 5** [Methods-2]**:** *What are the 12 cognitive function scores? If possible and this information is available the tests used should be added.*

**Author Response 5:** We have revised the sentence to clarify that the 12 cognitive function scores are derived from specific tests (e.g., CDRSB, ADAS11, MMSE, RAVLT, etc.) and indicated that detailed descriptions of these tests are provided in **S1 Table in S1 File** (**Lines 132-134 in Manuscript**).

**Comment 6** [Methods-3]**:** *The ROSMAP dataset was also obtained from ADNI or from somewhere else? as it is not clear too me.*

**Author Response 6:** To clarify, the ADNI dataset was obtained from the Alzheimer’s Disease Neuroimaging Initiative (http://adni.loni.usc.edu), and the ROSMAP dataset was obtained from the Synapse (https://www.synapse.org/Synapse:syn3219045) with appropriate data access permissions. The ROSMAP data was used in GWAS analyses to provide effect sizes for SNPs under different disease stages of the ADNI cohort. I have added this clarification to the Materials and Methods section (**Lines 138-142 in Manuscript**).

**Comment 7** [Methods-4]**:** *Line 126-127: It would be better to represent the following as a table.*

**Author Response 7:** We agree that this would enhance the clarity and readability of the information. We have revised the manuscript by replacing the text with a table titled “**Table 1.** Case Distribution in ADNI and ROSMAP Cohorts.” (**Line 159 in Manuscript**).

**Comment 8** [Methods-5]**:** *What tool was used for statistical analysis? R, excel, SPSS?*

**Author Response 8:** To clarify, all statistical analyses, including the application of the KNN algorithm for filling missing values and the Kruskal-Wallis test and chi-square test for group comparisons, were performed using the R software (version 4.3) (**Lines 156-158 in Manuscript**).

**Comment 9** [Methods-6]**:** *Line 177: What are the three ML integration strategies that were implemented?*

**Author Response 9:** We have revised the text to explicitly list the three integration methods (concatenation-based integration, transformation-based integration, and model-based integration) at the beginning of the section for improved readability (**Lines 222-224 in Manuscript**).

**Comment 10** [Methods-7]**:** *Was previously work done in order to decide the five ML methods that would be applied?*

**Author Response 10:** The choice of the five ML methods (KNN, LR, Bayes, SVM, and RF) was based on their fundamental nature and widespread use in the field of machine learning [1]. They represent diverse learning paradigms, including similarity-based (KNN), error-based (LR), probability-based (Bayes), kernel-based (SVM), and information-based (RF) approaches, making them suitable for evaluating the effectiveness of our model-based integration strategy.

Additionally, while advanced methods like XGBoost and AdaBoost are powerful, they often require extensive hyperparameter tuning and computational resources. Given our focus on evaluating fundamental integration strategies, we prioritized simpler yet representative methods to ensure a clear and interpretable comparison.

To address this point more explicitly in the manuscript, we have revised the relevant sentence in the Methods section as follows (**Lines 229-232 in Manuscript**):

“Finally, model-based integration uses separate classifiers for each omics data with five widely-used ML methods (KNN, LR, Bayes, SVM, and RF), selected for their diverse learning paradigms and fundamental nature in machine learning.”

Reference:

[1] Reel PS, Reel S, Pearson E, et al. Using machine learning approaches for multi-omics data analysis: A review. Biotechnology Advances. 2021;49:107739. doi:10.1016/j.biotechadv.2021.107739

**Comment 11** [Results-1]**:** *Add full names of the cognitive tests used under the table.*

**Author Response 11:** We have updated the manuscript to include the full names of the cognitive tests used under the table for clarity (**Lines 277-284 in Manuscript**).

**Comment 12** [Results-2]**:** *1KGP cohorts, why was this cohort not mentioned in the methods? unless I have missed it somewhere.*

**Author Response 12:** Thank you for your suggestion. We have supplemented the introduction of 1KGP data and the role of the LD panel in the PRS framework in the Materials and methods section (**Lines 146-150 and 185-189 in Manuscript**).

**Comment 13** [Results-3]**:** *A justification for the threshold values used in the QC process (why specific minor allele frequency or Hardy-Weinberg deviations thresholds were selected) would help readers understand the rationale for these decisions.*

**Author Response 13:** We have revised the manuscript to include the rationale behind the selection of these thresholds [1-4]. This addition aims to provide readers with a clearer understanding of the scientific basis for our QC decisions (**Lines 165-174 in Manuscript**).

Reference:

[1] Marees AT, De KH, Stringer S, et al. A tutorial on conducting genome‐wide association studies: Quality control and statistical analysis. International journal of methods in psychiatric research. 2018;27(2):e1608. doi:10.1002/mpr.1608

[2] Chang CC, Chow CC, Tellier LC, et al. Second-generation PLINK: rising to the challenge of larger and richer datasets. Gigascience. 2015 Dec;4(1):s13742-015. doi:10.1186/s13742-015-0047-8

[3] Wigginton JE, Cutler DJ, Abecasis GR. A note on exact tests of Hardy-Weinberg equilibrium. The American Journal of Human Genetics. 2005 May 1;76(5):887-93. doi:10.1086/429864

[4] Loh PR, Tucker G, Bulik-Sullivan BK, et al. Efficient Bayesian mixed-model analysis increases association power in large cohorts. Nature genetics. 2015 Mar;47(3):284-90. doi:10.1038/ng.3190

**Comment 14** [Results-4]**:** *The comparison between the four PRS models (P+T, LDpred, PRS-CS, and lassosum) is clear. However, a bit more detail on the algorithmic differences of each model would benefit readers who may not be familiar with these methods. For instance, what makes LDpred particularly suitable for this kind of analysis compared to PRS-CS or P+T?*

**Author Response 14:** We fully concur with this perspective and have supplemented in the manuscript the rationale for why LDpred is particularly suitable for this kind of analysis (**Lines 364-372 in Manuscript**):

“From the perspective of shrinkage mechanisms, P+T retains significant SNPs through hard thresholding but loses weak-effect signals, while PRS-CS employs global continuous shrinkage that excessively compresses moderate-effect variants. Lasso-type methods like lassosum analyze only partial SNPs in high-LD regions through regularization. In contrast, LDpred achieves localized adaptive shrinkage by explicitly modeling LD structures, which not only coordinates the adjustment of SNP effect sizes within high-LD regions but also preserves subthreshold genetic signals, thereby demonstrating higher variance explained and classification accuracy in AD risk prediction.”

**Comment 15** [Results-5]**:** *AUC analysis is very informative, but it would be helpful to state if improvements are statistically significant (or not), and whether the differences between models (e.g., LDpred vs. lassosum) are meaningful in a clinical context. Adding some statistical tests (e.g., pairwise comparison of AUCs) would provide stronger evidence.*

**Author Response 15:** We sincerely appreciate your suggestion to evaluate the statistical significance of AUC differences between models. However, after careful consideration, we decided not to perform formal hypothesis testing for the following reasons:

(1) Insufficient statistical power. Nonparametric tests (e.g., the Wilcoxon test) have very low sensitivity at n=5 and are difficult to detect true differences (prone to false negatives). Parametric tests (e.g., the t-parameter) require that the data be approximately normally distributed, but n=5 is not a reliable test for normality.
(2) The *p*-value is difficult to interpret. With small samples, *p*-values are very sensitive to extreme values, which can result in significant results by chance (false positives) or mask true differences (false negatives).
(3) Additionally, the most works of polygenic risk score performance evaluations exclusively utilize AUC metrics without incorporating p-values in their assessments [1-3].

So we choose to list the mean AUC of each group to compare the differences of the four methods, as shown in **Table 3** (**Lines 353-354 in Manuscript**).

Reference:

[1] Ni G, Zeng J, Revez JA, et al. A comparison of ten polygenic score methods for psychiatric disorders applied across multiple cohorts. Biological psychiatry. 2021 Nov 1;90(9):611-20.
[2] Thompson DJ, Wells D, Selzam S, et al. A systematic evaluation of the performance and properties of the UK Biobank Polygenic Risk Score (PRS) Release. Plos one. 2024 Sep 18;19(9):e0307270.
[3] Lennon NJ, Kottyan LC, Kachulis C, et al. Selection, optimization and validation of ten chronic disease polygenic risk scores for clinical implementation in diverse US populations. Nature medicine. 2024 Feb;30(2):480-7.

**Comment 16** [Results-6]**:** *The results suggest that PRS models are particularly useful in distinguishing CN from AD, but the manuscript could benefit from a deeper exploration of why this is the case.*

**Author Response 16:** We sincerely appreciate your insightful comment regarding the need to further explore the underlying mechanisms behind PRS’s discriminative performance between CN and AD. In response to your suggestion, we have expanded our discussion in the Results to provide a more comprehensive explanation. The additional explanation in the original article is as follows (**Lines 357-362 in Manuscript**):

“This excellent ability of PRS in distinguishing CN from AD stems from the polygenic genetic nature of AD. As a highly inherited disease, AD is driven by both high-effect variants such as *APOE-ε4* and numerous small-effect loci. PRS integrates these genetic effects to give AD patients a significantly higher cumulative risk than the CN population with a protective or neutral genetic background.”

**Comment 17** [Results-7]**:** *The authors report KS values and p-values for the linear fits based on LDpred PRS, but a more thorough interpretation of the statistical significance in the context of AD stages would be helpful.*

**Author Response 17:** In response, we have conducted a more thorough interpretation of the statistical significance within the context of AD progression stages. Specifically, we have: (1) elaborated on the respective clinical implications of both KS values and p-values, (2) provided detailed exemplification using ADAS13 as a representative cognitive metric, and (3) ultimately demonstrated the robust correlation between LDpred-derived PRS and cognitive function phenotypes (**Lines 376-393 in Manuscript**).

**Comment 18** [Results-8]**:** *The description of methylation data processing using ChAMP and feature selection via Fisher Score and entropy filtering is rigorous, but I would encourage the authors to provide more detail on the thresholds used for quartile-based feature selection. How were these thresholds determined, and do the results vary with different thresholds? This will help readers assess the robustness of your feature selection approach.*

**Author Response 18:** We appreciate the constructive suggestions on our feature selection methodology. In response, we have provided a detailed rationale for selecting quartile-based thresholds in both Fisher Score and entropy filtering approaches (**Lines 404-414 in Manuscript**).

The approximately normal distribution of Fisher Scores (**S5-S7 Figs in S1 File**) makes quartile thresholds statistically appropriate. In the Fisher Score-filtered probe set, we further applied entropy-based filtering to remove probes with zero information gain. A comparison showed that, across the three groups, the number of probes with information gain greater than zero was similar to the number of probes within the top 25%–100% quartile range (**S2 Table in S1 File**). Given the minimal differences between the two methods, we adopted the 25% threshold for both to streamline the process and maintain consistency.

**Comment 19** [Results-9]**:** *The AD-GCN as a novel approach for multi-omics integration is particularly interesting. However, the technical description of the graph convolutional network (GCN) could benefit from additional explanation. Specifically, how are cognitive scores incorporated into the graph model? Are these scores treated as node features or edge weights? Further clarification on how the cognitive scores are integrated into the graph structure would help readers better understand this innovative aspect of the model.*

**Author Response 19:** As suggested, we have clarified the integration of cognitive scores in the revised manuscript. Specifically, the AD-GCN architecture incorporates the similarity of cognitive scores between samples as edge weights in the graph structure, while the nodes represent integrated multi-omics features (BDI factors, polygenic risk scores, and methylation data). This design allows the model to simultaneously capture: (1) biological relationships through node features, and (2) clinical-functional associations via cognitive-weighted edges (**Lines 468-473 in Manuscript**).

**Comment 20** [Discussion-1]**:** *The authors should mention the benefits of this approach to AD and how it may be applied to other NDs as well, as this approach is novel and holds great research potential.*

**Author Response 20:** We sincerely appreciate the valuable suggestion regarding the broader implications of our AD-GCN framework. We have explicitly highlighted the benefits of this approach to AD and how it may be applied to other NDs (**Line 571-593 in Manuscript**):

“The AD-GCN framework demonstrates superior diagnostic accuracy compared to conventional machine learning integration methods by effectively capturing complex relationships among three readily accessible and cost-effective omics data types: clinical parameters, genetic variants, and methylation profiles. Specifically, our approach utilizes: (1) routine clinical assessments obtainable, (2) a focused panel of 1,322 high-risked SNPs, and (3) a minimal set of 10 methylation probes. This optimized panel design offers significant advantages over WGS or EPIC microarray approaches, achieving comparable diagnostic efficacy while substantially reducing both technical complexity and implementation costs. In clinical application, AD-GCN can achieve patient diagnosis through standardized data acquisition process: Firstly, the clinical data were obtained based on the structured doctor interview scale and the electronic medical record system. Secondly, the peripheral blood of the patient was collected, and then the gene variants and methylation markers were detected by the customized detection panel.

As an innovative multi-omics integration framework, AD-GCN has potential application value for polygenic neurological disorders (NDs), taking Parkinson’s disease (PD) as an example: The graph network structure of AD-GCN can naturally accommodate PD-related multi-omics data (such as motor symptom scores, GWAS significant loci, and blood methylation marks) to achieve accurate diagnosis of PD. Furthermore, the analytical framework of AD-GCN offers potential for adaptation to investigate other complex NDs. Such applications could facilitate the identification of disease-specific multi-omics risk factors, which in turn could inform the development of more targeted, accurate, and potentially cost-effective panels for early risk screening ultimately advancing precision diagnosis and management for these complex disorders.”

**Comment 21** [Discussion-2]**:** *The authors should mention the limitations of their work.*

**Author Response 21:** Thank you for your suggestion. At the end of the Discussion, we have added the limitations of the research work (**Line 594-616 in Manuscript**), including: (1) Expanding Population Diversity; (2) Incorporating Additional Omics Modalities; (3) Integrating Longitudinal Data for Dynamic Modeling.

**Comment 22** [General comments-1]**:** *Add full name followed by abbreviation. e.g. line 99.*

**Author Response 22:** Thank you for your suggestion. As per your comment, we have revised the manuscript to ensure that “polygenic risk scores (PRS)” is used at its first mention (**Line 118 in Manuscript**).

**Comment 23** [General comments-2]**:** *Add full name and links of databases. Also add references to the the database if there is.*

**Author Response 23:** Thank you for your suggestion to add the full names, links, and references for the databases used in our study. We have revised the manuscript to include this information (**Lines 130, 138, and 146 in Manuscript**).

**Comment 24** [General comments-3]**:** *Sample Homogeneity: The use of only Caucasian participants is understandable, but there should be a more thorough discussion of potential limitations this introduces, particularly in terms of generalizability to other ethnic groups. Given that AD and related diseases have genetic heterogeneity across populations.*

**Author Response 24:** We acknowledge this limitation and have expanded our discussion regarding the constraints of exclusively using White participants in the manuscript (**Lines 594-603 in Manuscript**):

“The study cohort predominantly comprised individuals of European ancestry. This limits the generalizability of our findings, as genetic background, environmental exposures, and lifestyle factors influencing AD etiology and biomarker expression can differ substantially across ethnic groups. Consequently, the clinical applicability of the current model requires validation and potentially optimization for non-European populations. Future investigations should prioritize the inclusion of diverse cohorts representing multi-ethnic population. Such expansion will facilitate comparative analyses of genetic background across diverse ancestries, potentially revealing ancestry-specific disease mechanisms.”

**Comment 25** [General comments-4]**:** *The figures should be made into a higher resolution.*

**Author Response 25:** Following the suggestion, we have systematically upgraded all figures (both in the main manuscript and supplementary materials) to 300 dpi resolution to ensure optimal image quality.

**Reviewer #3**

**Comment 1:** *Abbreviations (e.g., BDI, MCC) should be defined at first mention.*

**Author Response 1:** We appreciate your inquiry regarding the abbreviations. BDI stands for Basic Demographic Information, encompassing four key characteristics: age, sex, educational level, and marital status. MCC refers to Matthews Correlation Coefficient. For precise locations, these terms are defined in the Materials and methods section (BDI: **Line 132 in Manuscript**; MCC: **Lines 234-235 in Manuscript**) and consistently used throughout the manuscript.

**Comment 2:** *The Introduction section provides a comprehensive background on AD and multi-omics data integration. However, more details on existing graph convolutional network approaches for similar applications could be included to position the current work more clearly within the field.*

**Author Response 2:** We sincerely appreciate your suggestion. In response, we have significantly expanded the Introduction section to incorporate existing graph convolutional network approaches for similar applications, with the following substantive additions (**Lines 82-104 in Manuscript**):

“The fusion of multi-omics data is gradually becoming a cutting-edge approach for the precise diagnosis of AD [8-12]. Early multi-omics integration methods were primarily based on statistics and traditional machine learning (ML) models, encompassing three principal strategies: feature concatenation-based, transformation-based, and model-based integration. Specifically, concatenation-based integration directly combines multi-modal feature vectors as classifier inputs; transformation-based integration performs dimensionality reduction/feature transformation prior to fusion; model-based integration independently trains single-omics models before aggregating outputs. However, these conventional methods fail to account for inter-omics correlations, potentially introducing modality-specific biases. Recent advances have witnessed the emergence of graph convolutional networks (GCN) as powerful deep learning tools for biomedical multi-omics classification, demonstrating superior capability in modeling complex network-structured data relationships [13,14]. Schulte-Sasse et al. [15] developed an interpretable GCN framework integrating pan-cancer multi-omics data (mutations, copy number variations, DNA methylation, and gene expression) with protein-protein interaction networks for cancer gene prediction. Wang et al. [16] leveraged GCNs and sample similarity metrics to exploit cross-omics label-space correlations for tumor classification. These approaches effectively leverage the graph-structured properties of GCNs, which (1) represent inter-omics topological relationships as graph networks and (2) elucidate cross-omics feature dependencies through adaptive learning of edge weights. Consequently, the development of GCN-based diagnostic models capable of comprehensively integrating multi-omics data while exploiting inter-omics interactions presents a highly promising research direction.”

**Comment 3:** *LDpred outperforms other PRS methods, but the manuscript lacks a discussion of why LDpred is better suited for AD risk modeling compared to alternatives like PRS-CS.*

**Author Response 3:** We totally agree with this perspective and expanded the manuscript to elaborate on why LDpred is particularly suitable for this type of analysis (**Lines 364-372 in Manuscript**):

“From the perspective of shrinkage mechanisms, P+T retains significant SNPs through hard thresholding but loses weak-effect signals, while PRS-CS employs global continuous shrinkage that excessively compresses moderate-effect variants. Lasso-type methods like lassosum analyze only partial SNPs in high LD regions through regularization. In contrast, LDpred achieves localized adaptive shrinkage by explicitly modeling LD structures, which not only coordinates the adjustment of SNP effect sizes within high-LD regions but also preserves subthreshold genetic signals, thereby demonstrating higher variance explained and classification accuracy in AD risk prediction.”

**Comment 4:** *Validate Generalizability: Test AD-GCN on ROSMAP or another independent cohort.*

**Author Response 4:** We sincerely appreciate the valuable suggestion regarding the need for external validation on independent cohort, and we fully acknowledge the importance of demonstrating generalizability across different populations. However, we regret to report that due to current data accessibility limitations, we were unable to perform validation on external datasets as our study required samples with concurrent availability of comprehensive clinical profiles (including 16 demographic and cognitive features), whole genomic sequence (WGS) data, and DNA methylation profiles. An extensive search of Alzheimer’s disease repositories (ADNI, Synapse, GEO) did not yield existing cohorts that contained the requisite combination of clinical, WGS, and DNA methylation data essential for our analysis Furthermore, access to large-scale resources such as the UK Biobank was not feasible under current data access protocols. Acknowledging this limitation, we implemented rigorous internal 5-fold cross-validation with repeated iterations, stratified sampling to preserve class distributions (**Fig 11**), and conducted comprehensive ablation studies (**Lines 255-262 in Manuscript**) to assess feature contributions and model stability. These robust internal validation methods aim to ensure the reliability of our findings within the scope of the analyzed dataset. Future work will prioritize external validation when suitable multi-omics datasets become accessible.

**Comment 5:** *The Discussion overemphasizes technical contributions; a deeper exploration of biological insights and future research directions could be further expanded.*

**Author Response 5:** We sincerely appreciate your constructive feedback regarding the Discussion section. In response to your suggestion, firstly we have enhanced biological interpretation. We have added detailed analysis of how the identified genetic variants (e.g., *APOE*, *TOMM40*) and methylation genes (e.g., *AACS*) may influence AD pathogenesis through specific biological pathways (**Lines 531-554 in Manuscript**). Then we have proposed directions for future studies, including: expanding cohort diversity (multi-ethnic sampling), leveraging longitudinal data to track AD progression biomarkers, and integrating additional omics layers (e.g., proteomics) (**Lines 594-616 in Manuscript**).

**Reviewer #4**

**Comment 1:** *Clarity in Structure – The Results and Methods sections are interwoven, making it challenging to follow the study’s flow. A clearer separation of these sections would improve readability and comprehension.*

**Author Response 1:** We appreciate the constructive suggestions on the manuscript structure. To avoid redundancy, we have refined the Results section to concentrate exclusively on how the feature selection and modeling approaches were operationalized with ADNI data, removing methodological reiterations that were thoroughly presented in the Materials and Methods.

**Comment 2:** *GWAS and PRS Methodology – The description of GWAS and polygenic risk score (PRS) analysis needs more clarity. Instead of performing GWAS on a relatively small dataset, the authors could consider leveraging publicly available GWAS summary statistics to enhance the robustness and interpretability of findings. Similarly, more details on the thresholds and criteria used in PRS calculations and random forest feature selection would improve reproducibility.*

**Author Response 2:** We sincerely appreciate your insightful suggestions. Regarding the application of GWAS and PRS methods, we fully acknowledge that utilizing larger-scale GWAS summary statistics could indeed enhance the robustness and biological interpretability of PRS calculations. However, this study did not directly employ publicly available GWAS summary statistics due to the following critical considerations:

(1) To ensure population structure consistency, we rigorously selected predominantly European-descent samples from the ADNI cohort. Existing publicly available GWAS data (e.g., IGAP) often include multi-regional and multi-ethnic populations, whose heterogeneity may introduce confounding effects from population structure and environmental factors, potentially compromising the specificity of our results.
(2) Our study design incorporates three key comparison groups (CN vs. MCI, MCI vs. AD, and CN vs. AD) that require specific control data support. However, currently accessible public GWAS summary statistics only cover CN vs. AD comparisons, which cannot comprehensively meet our analytical requirements.
(3) The large global data repositories (i.e., UK Biobank) contain large-scale genomic data from Caucasian populations, making it particularly suitable as a data source for GWAS descriptive statistics. However, we regrettably did not obtain access to this database.

To mitigate these constraints, we adopted a carefully considered alternative methodology: First, we extracted European-descent samples from the ROSMAP cohort and systematically compared them with the ADNI Caucasian samples through population structure analysis (**Fig 2**), excluding individuals with significant genetic background differences. Subsequently, we reclassified the ROSMAP cohort into three comparison groups according to our research needs, obtaining group-specific GWAS summary statistics to provide reliable baseline data for PRS calculations in the ADNI cohort. This strategy ensures both population genetic background consistency and meets the requirements for multiple group comparisons, thereby guaranteeing the reliability and biological significance of PRS results.

In response to your questions regarding thresholds and criteria for PRS calculations and random forest feature selection, we consistently opted for default parameters. Specifically, all threshold parameters in the PRS calculation process employed default model settings, and similarly, the random forest algorithm maintained its default parameter configuration. This conservative approach aims to prevent local optimization artifacts that may arise from excessive parameter tuning. While we recognize the importance of parameter optimization, we believe that using default parameters at this stage, based on methodological rigor considerations, can more objectively reflect the model’s true performance.

**Comment 3:** *Clinical Applicability – The Discussion section could be expanded to address how AD-GCN could be integrated into clinical practice and what potential limitations or challenges might arise in real-world applications.*

**Author Response 3:** Thank you for your suggestions. In the discussion section, we supplement how the AD-GCN model can be applied in the clinic, and discuss the limitations of this application. The specific contents are as follows (**Lines 579-583 and 594-603 in Manuscript**):

“In clinical application, AD-GCN can achieve patient diagnosis through standardized data acquisition process: Firstly, the clinical data were obtained based on the structured doctor interview scale and the electronic medical record system. Secondly, the peripheral blood of the patient was collected, and then the gene variants and methylation markers were detected by the customized detection panel.”

“the study cohort predominantly comprised individuals of European ancestry. This limits the generalizability of our findings, as genetic background, environmental exposures, and lifestyle factors influencing AD etiology and biomarker expression can differ substantially across ethnic groups. Consequently, the clinical applicability of the current model requires validation and potentially optimization for non-European populations. Future investigations should prioritize the inclusion of diverse cohorts representing multi-ethnic population. Such expansion will facilitate comparative analyses of genetic background across diverse ancestries, potentially revealing ancestry-specific disease mechanisms.”

**Comment 4:** *Some sentences are not very fluent and the figures are not very clear.*

**Author Response 4:** We sincerely appreciate your constructive suggestions regarding textual refinement and visual presentation. In response, we have conducted a comprehensive linguistic review of the manuscript, ensuring clarity and coherence throughout all sections. All graphical elements (including main manuscript and supplementary materials) have been systematically upgraded to 300 dpi resolution, guaranteeing publication-standard image quality.

**Reviewer #5**

**Comment 1:** *The authors developed a machine learning tool named AD-GCN for Alzheimer’s disease diagnosis using clinical data, genomic variants, and DNA methylation data. The biggest issue is there are only 35 AD cases that have all three types of omics data, such a small sample size is not enough for the model to extract features. In addition, CN cases were 6 times more than AD cases, and MCI cases were 10 times more than AD cases in the ADNI dataset. The sample size imbalance in these groups will cause super-control bias in the model prediction. The average AUC of all 5 methods in MCI vs. AD and CN vs. AD are lower than 0.75, the AUC of two methods in CN vs. AD even fluctuates around 0.5 (Figure 5). This means that the above models are useless for AD diagnosis. Although the author did many analyses and evaluations of these models, the insufficient sample size is the fatal flaw of the manuscript.*

**Author Response 1:** We sincerely appreciate your insightful comments regarding the sample size limitations and class imbalance in our study. Regarding the concern about the small AD cohort (n=35), we acknowledge that this poses challenges for feature extraction. However, our methodological pipeline was carefully designed to mitigate these limitations:

(1) For clinical data, we utilized 16 features including basic demographic information (BDI: age, sex, education level, and marital status) and 12 cognitive assessment scores, all obtained directly from raw data without additional feature extraction.
(2) For the genetic variant data (SNPs), given that the SNP matrix constitutes a high-dimensional, sparse binary matrix (encoded as 0/1/2), we did not perform sample balancing. Instead, we directly computed polygenic risk scores (PRS) for each comparison group and experimentally validated their suitability as representative features through five-fold cross-validation, assessing both the AUC and phenotypic interpretability of the PRS. Our results demonstrate that combining PRS with BDI (“C+P” strategy) consistently yields superior classification performance compared to using BDI alone (“C” strategy). This suggests that PRS may enhance predictive accuracy beyond what can be achieved with clinical features (BDI) independently. Furthermore, we observed that more severe dementia cases (with weaker cognitive function) exhibited higher PRS-indicated risk scores, confirming the phenotypic interpretability of this measure. Collectively, these findings indicate that PRS provides meaningful contributions to classification performance. Consequently, we selected the best-performing PRS model (LDpred) as the representative feature for genetic variants, which was subsequently incorporated into our AD-GCN diagnostic model construction.
(3) For DNA methylation data, which consists of a two-dimensional matrix with values ranging from 0 to 1, we implemented a boundary-based hybrid sampling method to address class imbalance, achieving low imbalance ratios across all three comparison groups. Subsequently, we applied filter and embedding methods for feature extraction, identifying the top 10 most representative methylation subsets for each group (**S7 Table in S1 File**). When the top 10 methylation probes combined with BDI and LDpred PRS (“C+P+M” strategy), the classification results (shown in the **Fig 10**) consistently outperformed those obtained using the “C+P” strategy (LDpred PRS+BDI).

Overall, both PRS and the top 10 methylation probes demonstrate an improvement in disease classification performance, indicating the rationality and scientific validity of using PRS and top 10 methylation features as representative biomarkers.

To mitigate potential prediction bias induced by class imbalance, we uniformly applied identical data balancing procedures prior to all classification experiments. Following this adjustment: For the MCI vs. AD comparison, sample sizes were modified from 345:35 to 344:125 (imbalance ratio = 2.75). For the CN vs. AD comparison, sample sizes were adjusted from 209:35 to 206:105 (imbalance ratio = 1.96). Both groups maintained imbalance ratios below 3.0, representing only mild class imbalance. The classification experiments on this basis could greatly reduce the bias caused by class imbalance.

Regarding the observed AUC values (all below 0.75 for MCI vs. AD and CN vs. AD comparisons, with two methods approaching 0.5 in CN vs. AD as shown in Figure 5), it should be noted that the PRS serves as just one component in our final AD prediction framework. While the standalone predictive performance of PRS appears limited, we demonstrate that the combined PRS+BDI model (“C+P” strategy) achieves superior AUC performance compared to BDI alone (**S8 Table in S1 File**); and further incorporation of the top 10 methylation probes yields additional improvement in AUC (“C+P+M” strategy). Taking model-based integration as an example, the AUC values were as follows:

(1) BDI alone (“C” strategy): 0.76 for MCI vs. AD, 0.79 for CN vs. AD;
(2) BDI + LDpred PRS (“C+P” strategy): 0.80 for MCI vs. AD, 0.87 for CN vs. AD;
(3) BDI + LDpred PRS + Top 10 methylation probes (“C+P+M” strategy): 0.88 for MCI vs. AD, 0.90 for CN vs. AD.

These progressive enhancements in predictive accuracy systematically validate both the rationality and comparative advantage of our multi-omics integration approach.

Moreover, the limited AD sample size (n=35) reflects our stringent inclusion criteria requiring all three omics data types and European ancestry to ensure sample homogeneity. Unfortunately, we were unable to access larger datasets such as UK Biobank. We would welcome the opportunity to incorporate additional multi-omics samples should they become available in future studies.

**Reviewer #6**

**Comment 1:** *Biological Interpretation: The study identifies key genetic (APOE, CR1, TOMM40) and epigenetic (AACS, VTRNA2-1, CYP26C1) markers, but their roles in AD pathogenesis are not discussed in depth. A more detailed interpretation would be valuable.*

**Author Response 1:** Thank you for your suggestion. We have added detailed analysis of how the identified genetic variants (e.g., *APOE*, *TOMM40*) and methylation genes (e.g., *AACS*) may influence AD pathogenesis through specific biological pathways (**Lines 531-554 in Manuscript**).

In fact, the primary focus of this study lies in developing diagnostic models for complex diseases like AD, with particular emphasis on investigating how different multi-omics data integration approaches affect disease diagnostic performance, rather than identifying and mechanistically characterizing novel AD biomarkers. Our work primarily contributes to the methodological advancement of computational approaches for disease diagnosis by systematically evaluating various omics data fusion strategies and their impacts on model accuracy, while the biological interpretation of specific biomarkers, though acknowledged, remains secondary to our central research objective of optimizing diagnostic modeling frameworks.

**Comment 2:** *Justification for Adjacency Matrix Construction: The authors use sample similarity and cognitive scores to build the adjacency matrix in AD-GCN. Could they clarify why this approach was chosen over other possible graph structures (e.g., biological pathway-driven networks)?*

**Author Response 2:** Thank you for your suggestion. The rationale for constructing the network structure based on sample similarity and cognitive scores is as follows: sample similarity represents a well-established approach for graph construction [1,2]. In our experimental design, we employed a dual strategy for edge formation: (1) selecting sample pairs with similarity scores exceeding 0.5 as primary connections, and (2) augmenting the edge set through KNN analysis based on spatial proximity.

Cognitive scores were incorporated because they reflect clinically meaningful variations in patients’ cognitive and behavioral functions, which may correlate with underlying genetic risk (SNP profiles) and methylation levels. As AD-GCN operates on a sample-as-node graph architecture, we leveraged cognitive similarity to strengthen inter-node relationships. The validity of this approach is supported by ablation studies (**S8 Fig in S1 File**).

We appreciate the insightful suggestion regarding biological pathway-driven networks. Indeed, integrating protein-protein interaction data or biological pathways into neural networks represents a promising direction for future research, which we plan to explore in subsequent studies. This could potentially enhance the biological interpretability of our model while maintaining its computational efficiency.

Reference:

[1] Li MY, He X, Zhu HB, et al. Study on Classification Method of Alzheimer's Disease Convolutional Neural Network Combined with Phenotypic Information. Chinese Journal of Biomedical Engineering. 2021;40(2):177-87.

[2] Ouyang D, Liang Y, Li L, et al. Integration of multi-omics data using adaptive graph learning and attention mechanism for patient classification and biomarker identification. Computers in biology and medicine. 2023 Sep 1;164:107303.

**Comment 3:** *Computational Efficiency: AD-GCN is shown to outperform traditional ML models in classification accuracy, but the manuscript does not discuss its computational requirements. Providing details on runtime, memory usage, and scalability would be helpful for researchers looking to apply this method.*

**Author Response 3:** Thank you for your suggestion. In the Results section, we have supplemented the comparative analysis of computational requirements between AD-GCN and traditional machine learning models, including runtime and memory usage. The specific content is (**Lines 480-492 in Manuscript**):

“Compared with model-based integration methods (taking model-based integration as an example), AD-GCN demonstrates a reasonable performance trade-off: its runtime is approximately 2.9× longer than conventional approaches (CN vs. MCI: 13.2s vs. 5.49s; MCI vs. AD: 11.2s vs. 3.99s; CN vs. AD: 13.5s vs. 3.9s), Peak memory consumption was 1.7× higher (CN vs. MCI: 302.14 MiB vs. 175.95 MiB; MCI vs. AD: 303.00 MiB vs. 179.60 MiB; CN vs. AD: 290.98 MiB vs. 168.36 MiB) (**S9 Table in S1 File**). This modest elevation in computational resource requirements is justifiable given the significant improvement in classification accuracy (e.g., +9% in CN vs. MCI, +4% in MCI vs. AD, and +4% in CN vs. AD), particularly when considering the inherent computational complexity of graph neural networks in processing intricate topological relationships.”

**Comment 4:** *Generalizability and Population Diversity: The study is based on ADNI and ROSMAP, which primarily include Caucasian participants. Have the authors considered validating the model on more diverse datasets?*

**Author Response 4:** We sincerely appreciate the valuable suggestion regarding the need for external validation on independent cohorts, and we fully acknowledge the importance of demonstrating generalizability across different populations. However, we regret to report that due to current data accessibility limitations, we were unable to perform validation on external datasets as our study required European samples with simultaneous availability of comprehensive clinical profiles (including 16 demographic and cognitive features), peripheral blood SNP data, and matching methylation profiles. Our extensive search of established Alzheimer’s disease repositories (ADNI, Synapse, GEO) failed to identify any existing cohorts that simultaneously encompass the complete triad of omics data modalities essential for our analysis. Regrettably, access to more comprehensive resources like UK Biobank remains beyond our current data acquisition capabilities. External validation in non-European populations requires caution. The current study cohort primarily consists of individuals of European ancestry, and the genetic backgrounds, environmental exposures, and lifestyle factors influencing AD etiology and biomarker expression may vary substantially across ethnic groups. In future investigations, we plan to incorporate diverse cohorts representing multi-ethnic populations to evaluate the generalizability of the AD-GCN model across different genetic backgrounds.

While we recognize this as a limitation, we have implemented rigorous 5-fold cross-validation with repeated iterations using different random seeds, employed stratified sampling to preserve class distributions, and conducted comprehensive ablation studies to maximize the robustness of our findings within these constraints. We hope that the methodological rigor of our internal validation approaches helps compensate for the lack of external validation at this stage, and we would be delighted to incorporate additional validation cohorts should suitable multi-omics datasets become accessible in future work.

**Comment 5:** *Reproducibility: The authors mention that their code is available on GitHub. Could they confirm whether preprocessing scripts and detailed environment configurations are included to allow full replication of results?*

**Author Response 5:** Thank you for your inquiry. We have carefully reviewed the code on GitHub and have supplemented comprehensive environment configuration details (in the “**00prepare**” folder). The detailed preprocessing scripts are now integrated within the data analysis workflow scripts rather than being stored separately. This revision ensures better reproducibility and clarity for users following our computational pipeline.
